# Enhancing Biopolyester Backbone Rigidity with an Asymmetric Furanic Monomer

**DOI:** 10.1021/acssuschemeng.5c07913

**Published:** 2025-10-08

**Authors:** Cristian P. Woroch, Bennett Addison, Alexandra Stovall, Erik Rognerud, Clarissa Lincoln, Joel Miscall, Gloria Rosetto, Matthew W. Kanan, Nicholas A. Rorrer, Gregg T. Beckham

**Affiliations:** † Department of Chemistry, 6429 Stanford University, 337 Campus Drive, Stanford, California 94305, United States; ‡ Renewable Resources and Enabling Sciences Center, 53405 National Renewable Energy Laboratory, Golden, Colorado 80401, United States

**Keywords:** furan polyester, performance-advantaged bioplastic, hydroxyester polycondensation, chain mobility, crystallization kinetics, molecular dynamics

## Abstract

Biobased furanic polyesters can exhibit performance advantages over petroleum-derived polyesters, primarily due to their rigid furan-containing backbones. Herein, we develop two strategies to polymerize methyl 5-hydroxymethyl furanoate to poly­(5-hydroxymethyl furanoate) (PHMF), a furan-based polyester with even greater backbone rigidity than poly­(ethylene furanoate). Thermal, spectroscopic, and computational investigations of PHMF alongside analogous furan-based and phenyl-based polyesters suggest that the high furan content of PHMF leads to its high glass transition temperature, slow crystallization kinetics, and low amorphous mobility. Molecular dynamics simulations suggest that while the backbone of PHMF is exceptionally rigid, its amorphous phase is denser than its phenyl analog due to noncovalent interchain interactions. Together, these results highlight how asymmetric furan-based monomers can modulate key properties in biobased polyesters.

## Graphical abstract

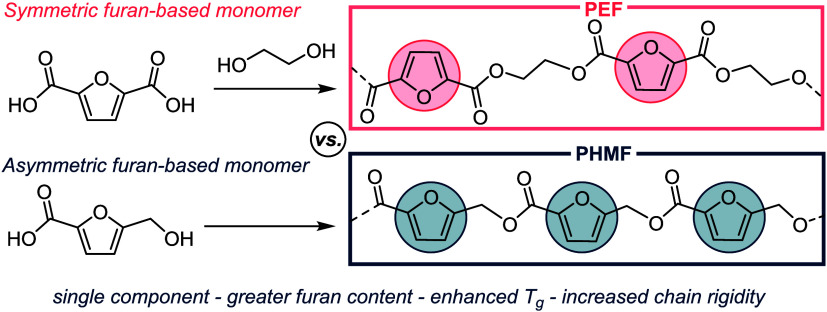

## Introduction

While more than 99% of plastic is currently produced from petroleum,^1^ plastics derived from biogenic feedstocks can be produced more sustainably than and exhibit performance advantages relative to petroleum-derived materials.^2^ For example, furan-based polyesters such as poly­(ethylene furanoate) (PEF) exhibit a higher glass transition temperature (*T*_g_), higher Young’s modulus, and superior gas barrier properties relative to petroleum-derived poly­(ethylene terephthalate) (PET) and have thus inspired considerable industrial efforts toward producing furanic polymers at scale.^3–12^ The enhanced properties of PEF have been attributed to the rigidity of its furan-containing backbone, which decreases chain mobility, increases the *T*_g_, and decreases gas permeability.^13–15^ Developing strategies to enhance rigidity in furan-based polyesters could enable further enhancement of their desirable properties and incentivize their adoption over petroleum-derived materials.

Furan-based polyesters are typically synthesized via polycondensation of furan-2,5-dicarboxylic acid (FDCA) with aliphatic diols.^3,9,16–28^ Since ethylene glycol is the shortest diol capable of polymerizing with FDCA,^18^ PEF achieves the highest furan content possible for this class of materials. Increasing the furan content within the polymer backbone is possible by polymerizing the asymmetric furan-based monomer 5-hydroxymethyl furoic acid (HMFA). With a similar structure to FDCA, HMFA is the simplest furan-based hydroxyacid and is commonly prepared by mild oxidation of fructose-derived 5-hydroxymethyl furfural (HMF) or hydroxymethylation of lignocellulose-derived furoic acid (FA).^29–37^ Like PEF, the polymerization of HMFA to poly­(5-hydroxymethyl furoic acid) (PHMF) yields a fully biobased polyester with a high furan backbone content. In principle, PHMF can be sourced from a single hydroxyacid monomer, thereby reducing the complexity for synthesis and chemical recycling. Despite these potential advantages, polycondensation of HMFA to poly­(5-hydroxymethyl furanoate) (PHMF) has seldom been reported.^35,38–41^ To date, the only reported method capable of synthesizing high-molar-mass PHMF (>10 kDa) involves cyclic oligomerization of HMF or HMFA followed by macrocyclic ring-opening polymerization.^41,42^

In this work, we report two polycondensation methods to synthesize PHMF from methyl 5-hydroxymethyl furanoate (MHMF) prepared via Fischer esterification of biomass-derived HMFA (Figure 1a). To understand the impact of the furan ring, we also synthesized the phenyl analogue of PHMF, poly­(*m*-hydroxymethyl benzoate) (PHMB). Using spectroscopic, thermomechanical, and computational methods, we compare the properties of these two hydroxyacid-derived polyesters to PET and PEF (Figure 1b). Together, these measurements suggest that the polymers, based on the asymmetric hydroxyacid repeat unit, have more rigid polymer backbones, resulting in reduced amorphous mobility and slower crystallization kinetics. In addition, compared to the phenyl-based polyesters, the furan-based polyesters exhibit higher glass transition temperatures (*T*_g_) and higher densities, which may contribute to advantageous thermal and gas barrier properties.^13^ As a furan-based polyester with an asymmetric repeat unit, our results indicate that PHMF exhibits a unique chain structure leading to its exceptionally high *T*_g_ and is worthy of further study.

**Figure 1:**
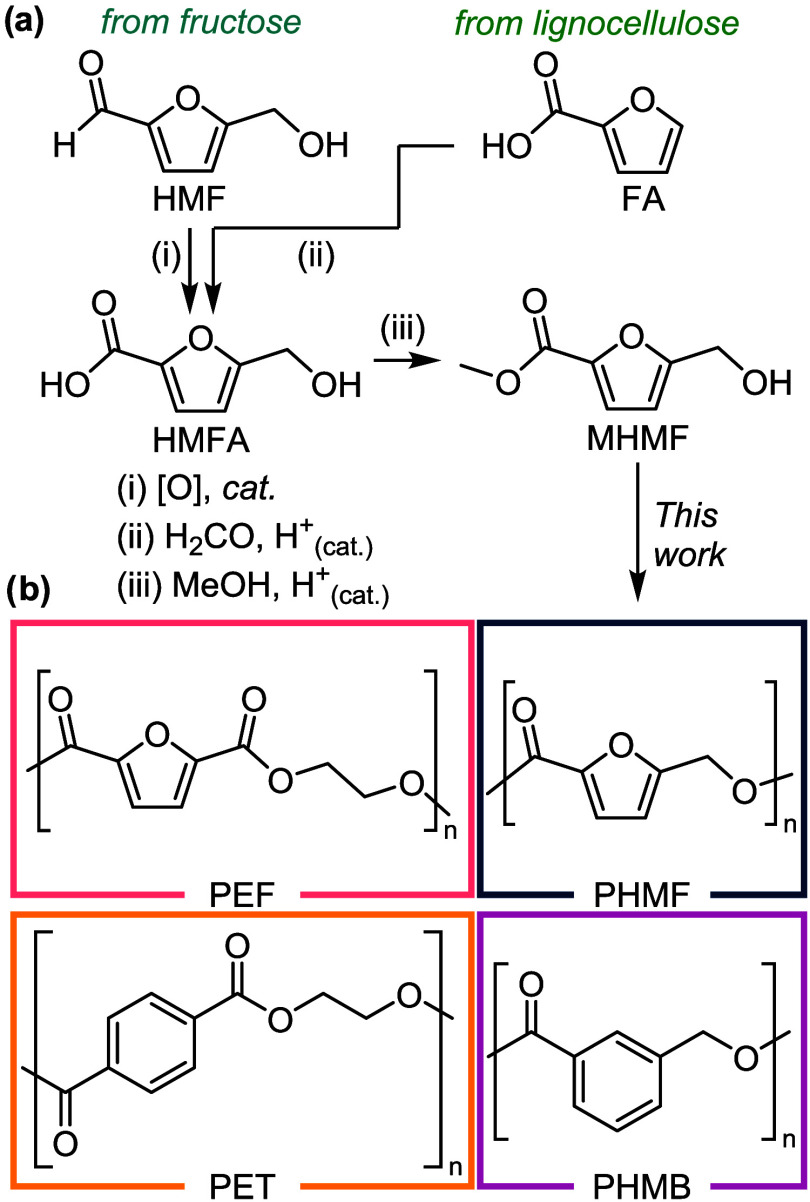
(a) Synthesis of PHMF from bio-derived sources via oxidation of HMF,^29–35^ hydroxymethylation of FA,^36,37^ and Fischer esterification of HMFA. (b) The chemical structures of the polyesters examined in this study.

## Results and Discussion

### Synthesis of PHMF via Melt and Solution-Based Methods

Previous reports exploring standard polycondensation to synthesize PHMF have yielded polymers with low average degrees of polymerization (DP < 20).^35,38–40^ We thus first sought to optimize a melt condensation method to directly polymerize MHMF to PHMF with a high degree of polymerization (DP > 100) without requiring stoichiometric reagents. A critical first step was obtaining high-purity MHMF. We observed that MHMF synthesized by Fischer esterification and purified via column chromatography yielded an oil that was insufficiently pure to produce high-molar-mass PHMF. We determined that recrystallization of MHMF in diethyl ether or cyclopentyl methyl ether at −20 °C overnight converted MHMF from an oil to an off-white solid and improved monomer purity to >99.9% as determined by a van’t Hoff analysis using differential scanning calorimetry (DSC, Figure S1, see Supporting Information (SI)).^43^

Several catalysts were screened in small-scale polycondensation experiments, including Sb_2_O_3_, Zn­(OAc)_2_, and Ti­(*i*-PrO)_4_, with (Oct)_2_SnO exhibiting the highest yield and conversion (Table S1). While elevated temperatures of 180 °C resulted in high polymer yields in a shorter reaction time (4 h), a reduced reaction temperature of 140 °C over a longer reaction time (9–18 h) was preferred because it resulted in minimal discoloration (Figure S2). To confirm the structure of the synthesized material, PHMF was characterized via several NMR spectroscopy methods (^1^H, ^13^C, heteronuclear single quantum coherence (HSQC), heteronuclear multiple bond correlation (HMBC), and correlation spectroscopy (COSY)) and matrix-assisted laser desorption ionization time-of-flight (MALDI-TOF) mass spectrometry (Figures S3–S8). End-groups were identified by ^1^H NMR spectroscopy, and observed *m*/*z* ratios were consistent with methyl-terminated PHMF chains, suggesting this polycondensation method yields linear PHMF polyester.

The polycondensation method developed at the milligram scale was subsequently scaled to the gram scale. However, a single temperature step without agitation was insufficient to produce high-molar-mass PHMF in a good yield (Table S2). Therefore, a multistep procedure with temperature, atmospheric, and mixing control was developed by adapting previous methods used to synthesize PET and PEF (see SI, Figure S9).^12,44^ The highest yields were obtained when the initial polycondensation step was performed at a moderate temperature over a long period of time (140 °C, 18 h), which reduced unwanted monomer volatilization. Polycondensation of MHMF at a 5 g scale using the optimized multistep procedure produced PHMF at 73% yield. The gel permeation chromatography (GPC)-measured number-average molar mass (*M*_n,GPC_) and dispersity (*Đ*) of PHMF synthesized by the optimized method were found to be 13.7 kDa and 2.3, respectively.

To obtain higher-molar-mass PHMF, a postcondensation step was investigated. After purification through dissolution and precipitation, the polymer was mixed with additional catalyst and heated in a vial block set to 190 °C under high vacuum. This secondary postpolycondensation step was found to increase the number-average molar mass of PHMF substantially (*M*_n,GPC_ = 48.8 kDa, *Đ* = 1.1) (Figure 2a). We hypothesize that this additional step facilitates further melting and condensation of shorter polymer chains without significantly impacting longer chains, thereby increasing average molar mass, while narrowing the dispersity.

**Figure 2:**
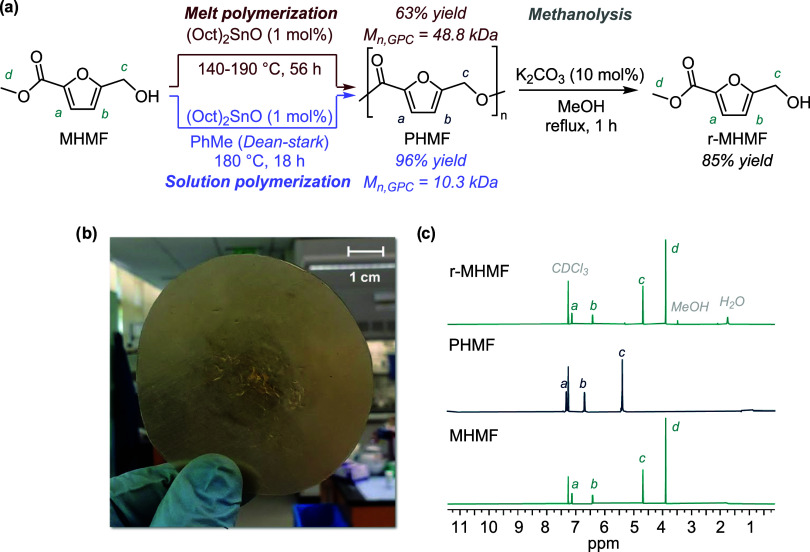
Synthesis and deconstruction of PHMF. (a) Solution- and melt-based polymerization and methanolysis depolymerization of PHMF. (b) Melt-pressed film fabricated from solution-polymerized PHMF. (c) ^1^H NMR spectra of (from bottom to top) virgin MHMF monomer, purified PHMF polymer, and recovered r-MHMF monomer following methanolysis (CDCl_3_/trifluoroacetic acid (TFA), 400 MHz).

The multistage polycondensation and postcondensation method described above produces high-molar-mass PHMF, but results in beige material which could not be fabricated into robust films (Figure S10a). Under elevated temperature and concentration conditions, furan-containing molecules are known to undergo a series of deleterious side reactions, including the formation of humins.^45^ Polycondensation in a refluxing solvent with a condensate trap has previously been shown to be an effective strategy to improve polymerization yield and reduce side reactions.^46^ Over the course of the reaction, the insoluble polymer precipitates and adheres to the bottom of the flask. The solution-based polycondensation strategy significantly improved the yield (>90%) and reduced the discoloration of the resulting PHMF (Figure S10b). While the resulting polymer had lower molar mass (*M*_n,GPC_ = 10.3 kDa, *Đ* = 1.7), PHMF synthesized using this method could be pressed into large, albeit brittle films (Figure 2b).

### Depolymerization of PHMF

Considering a polymer's end of life is critical when designing a new plastic material. The depolymerization of PHMF via methanolysis was selected as a promising chemical recycling strategy because of its moderate solvolytic activity, ability to produce an easily repolymerized methyl ester, and comparatively high tolerance of contamination.^47,48^ Unlike diacid-derived polyesters, hydroxyacid-derived polyesters like PHMF consist of a single monomer, thereby simplifying postdeconstruction separations. Chemical recycling of PHMF was initially performed on a small scale to screen viable conditions (Table S4). Both Zn­(OAc)_2_ and K_2_CO_3_ were identified as viable catalysts for methanolysis.^49,50^ By refluxing PHMF in methanol with a 10 mol% loading of K 2 CO 3 for 1 h, the polymer was completely converted to MHMF with no observed side products by ^1^H NMR spectroscopy (Figure 2c). Optimal conditions were replicated with 1 g of PHMF and resulted in quantitative conversion of the polymer. MHMF was isolated in 85% yield after drying the crude recyclate and extracting the organic monomers using ethyl acetate.

### Comparing the Thermal Properties of PHMF to Related Polyesters

For comparison to commercially-relevant polyesters, previously reported methods were adapted to synthesize PET (*M*_n,GPC_ = 21.7 kDa, *Đ* = 2.4) and PEF (*M*_n,GPC_ = 15.7 kDa, *Đ* = 3.4).^12,44^ Since biobased furan-based polyesters are heralded as potential replacements for petroleum-based phenyl polyesters, it is critical to understand how the polymer structure is impacted by the furan and phenyl moieties. Therefore, PHMB was synthesized to serve as a phenyl analog to PHMF. Melt polymerization of *m*-hydroxymethyl benzoate (MHMB) with 0.5 mol% (Oct)_2_SnO yielded 90% of the desired PHMB product (*M*_n,GPC_ = 7.0 kDa, *Đ* = 2.7). The solution-based polymerization of MHMB with 1 mol% (Oct)_2_SnO also yielded the desired PHMB product (*M*_n,GPC_ = 9.9 kDa, *Đ* = 2.4). After synthesis, each material was analyzed by ^1^H NMR spectroscopy for structural confirmation and GPC to characterize the molar mass distribution (Figures S12–S20). Since PHMB has not been fully characterized in previous literature,^51–56^ additional NMR spectroscopy (^13^C, HSQC, HMBC, and COSY) and MALDI-TOF mass spectrometry measurements were used to confirm its structure (Figures S15–S19). We note that the polyester syntheses were complicated by glycol dimerization during the synthesis of PET and PEF and poor molar mass control, thus limiting our ability to synthesize polyesters with identical degrees of polymerization (DP). Since differences in molar mass and polymerization method can significantly contribute to differences in observed thermal properties,^57^ only polyesters synthesized from the melt polycondensation with moderate degrees of polymerization were analyzed further (Table S5).

Each polyester was initially analyzed by DSC to examine its thermal phase transitions. In the first DSC cycle, every polyester displayed an endothermic event, indicative of a *T*_m_. PET displayed the highest melting transition (*T*_m_ = 249 °C), followed by PEF (*T*_m_ = 215 °C), PHMF (*T*_m_ = 190 °C), and PHMB (*T*_m_ = 188 °C). Of the four polyesters examined, only PET displayed a clear crystallization event during cooling. On the second heating cycle, PET and PEF exhibited melting transitions, whereas PHMF and PHMB did not. All polyesters exhibited a *T*_g_ on the second cycle, though in the case of PEF and PET, the magnitude of the transitions was very weak. Of the four polyesters examined, PHMF exhibited the highest glass transition temperature (*T*_g,DSC_ = 83 °C) followed by PEF (*T*_g,DSC_ = 80 °C), PET (*T*_g,DSC_ = 74 °C), and PHMB (*T*_g,DSC_ = 67 °C). A similar trend in *T*_g_ is observed when films of each polyester are characterized by dynamic mechanical analysis (DMA) with a cycle frequency of 1 Hz (Figures 3 and S21). The glass transition temperature measured by the peak of the tan δ curve for PHMF is the highest (*T*_g,tan δ_ = 112 °C) followed by PEF (*T*_g,tan δ_ = 90 °C), PET (*T*_g,tan δ_ = 85 °C), and PHMB (*T*_g,tan δ_ = 77 °C). While the absolute values of *T*_g,tan δ_ diverge from those of *T*_g,DSC_ due to time–temperature superposition,^58^ the trend in the *T*_g_ values is consistent. In addition, both PEF and PHMF exhibit secondary peaks in the tan δ curve consistent with cold crystallization, further suggesting the rigidity of the furan unit slows crystallization.^59^ Furthermore, the magnitude of the maximum of tan δ indicates that the furan polyesters have higher dampening ratios than the phenyl polyesters.^59,60^ Lastly, thermogravimetric analysis (TGA) was used to determine the thermal stability of each material. The thermal decomposition onset temperature (*T*_d,5%_) was highest for PET (*T*_d,5%_ = 395 °C), followed by PHMB (*T*_d,5%_ = 379 °C), PEF (*T*_d,5%_ = 358 °C), and PHMF (*T*_d,5%_ = 312 °C), suggesting the furan moiety reduces thermal stability.

**Figure 3:**
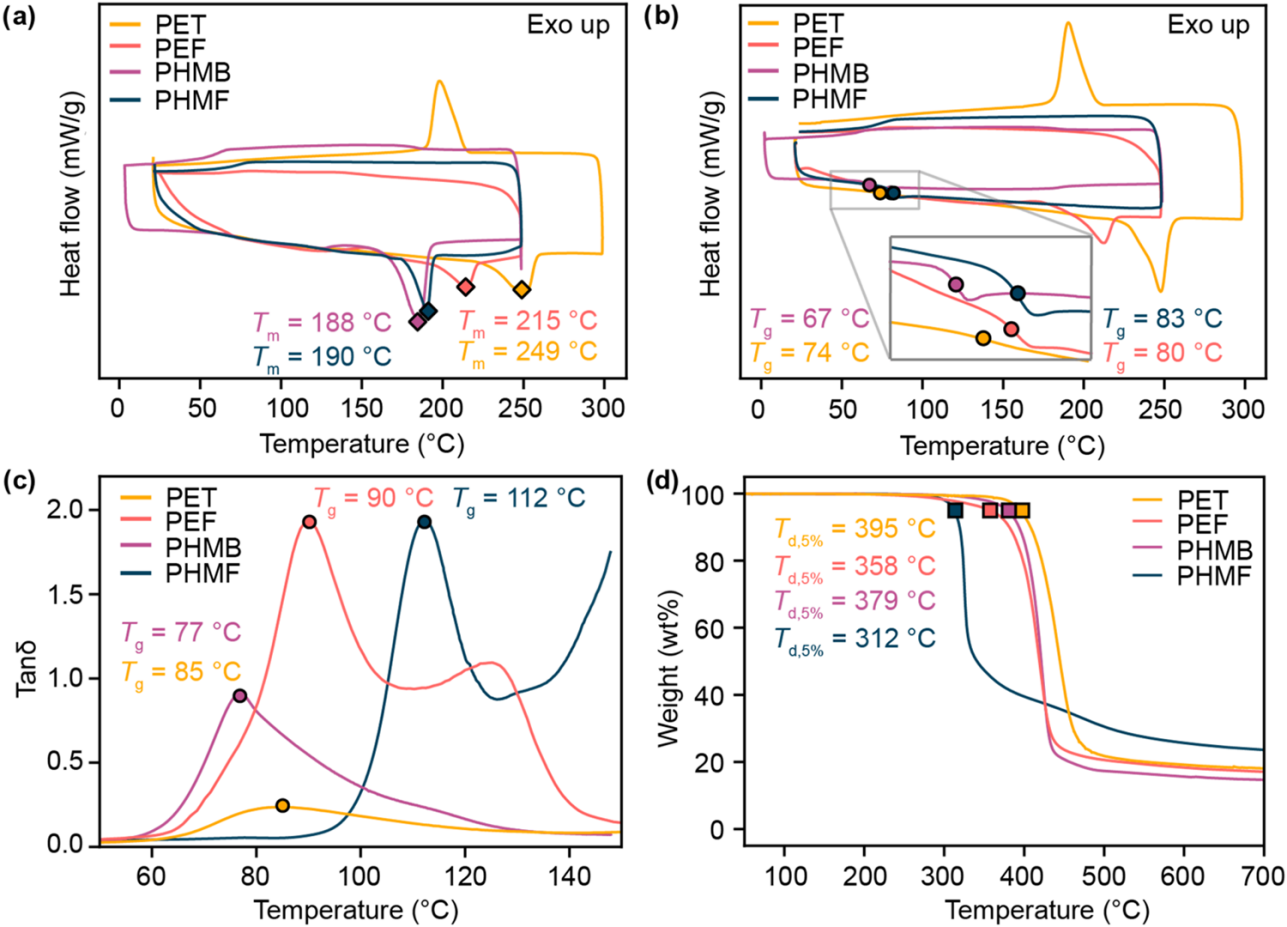
Thermal analysis of polyesters including (a) the first cycle of DSC (10 °C/min) to identify *T*_m_, (b) the second cycle of DSC (10 °C/min) to identify *T*_g,DSC_, (c) DMA (5 °C/min, 1 Hz, 0.1% strain) to identify *T*_g,tan δ_ and the maximum of tan δ, and (d) TGA (10 °C/min) to identify *T*_d,5%_.

Thermal phase transitions are governed by chain dynamics and intermolecular interactions and thus provide insights into molecular-scale behavior. The *T*_g_, for example, is correlated to chain rigidity and inversely correlated to polymer free volume.^58^ We observe that *T*_g_ is significantly higher for the furan-based polymers—PEF and PHMF—relative to their phenyl-based counterparts—PET and PHMB. Further, by both DSC and DMA, PHMF exhibits a higher *T*_g_ than PEF, which we hypothesize results from additional rigidity due to the absence of contiguous methylene groups in the polymer backbone. Since previous work on PET, PEF, and PHMF report only minor differences in *T*_g_ between samples exceeding 10 kDa,^41,42,61,62^ we believe the observed differences cannot be explained exclusively by differences in DP. Melting temperature, in addition, is correlated to the stability of crystalline packing for a given polymer.^63^ Thus, we observe that the polyesters from the asymmetric monomers, PHMF and PHMB, form less stable crystalline regions than those from the symmetric monomers, PET and PEF.

### Investigating Polyester Chain Dynamics through Isothermal Crystallization

While PET and PEF exhibit melting events in the second DSC cycle, PHMF and PHMB do not, suggesting that the crystallization rate of these polyesters is significantly slower. In polymeric systems, the rate of crystallization is kinetically controlled and determined by numerous molecular factors, including interchain interactions, chain mobility, and free volume.^64^ Further, understanding the crystallization of polymeric materials is critical for practical utility, as the rate and extent of crystallinity determine processability and mechanical properties.^65,66^

Crystallization of a polymer is known to occur fastest at an intermediate temperature between the *T*_g_ and the *T*_m_.^64^ To estimate the maximum rate of crystallization, each polyester was heated above *T*_m_ and then rapidly cooled and held at a crystallization temperature (*T*_c_) between *T*_g_ and *T*_m_ during which the heat of crystallization (*Q*_c_) was measured. The heat released during crystallization was integrated, normalized, and fit via Avrami analysis to obtain the Avrami exponent (*n*), Avrami rate constant (*k*), and crystallization half-life (*t*_1/2_) (eq S1 and Figure 4a,b). Of the four polyesters, PET was found to crystallize the fastest (*t*_1/2_ = 147 s), followed by PEF (*t*_1/2_ = 248 s), PHMF (*t*_1/2_ = 912 s), and PHMB (*t*_1/2_ = 4469 s). The *t*_1/2_ values correlated with *n*, but they inversely correlated with *k*. Together, these data indicate that the polyesters based on asymmetric repeat units crystallize more slowly than those with symmetric repeat units; however, the *n* values suggest that crystallite growth of the asymmetric polyesters occurs by a more three-dimensional expansion than that of the symmetric polyesters. In addition, the slower crystallization rate of PHMB relative to PHMF indicates the furan ring exhibits an accelerating effect on crystallization. Previous work has observed an inverse relationship between molar mass and rate of crystallization,^57,67–69^ suggesting the difference in crystallization rate between PHMF and PHMB may be even greater for samples of identical degrees of polymerization.

**Figure 4:**
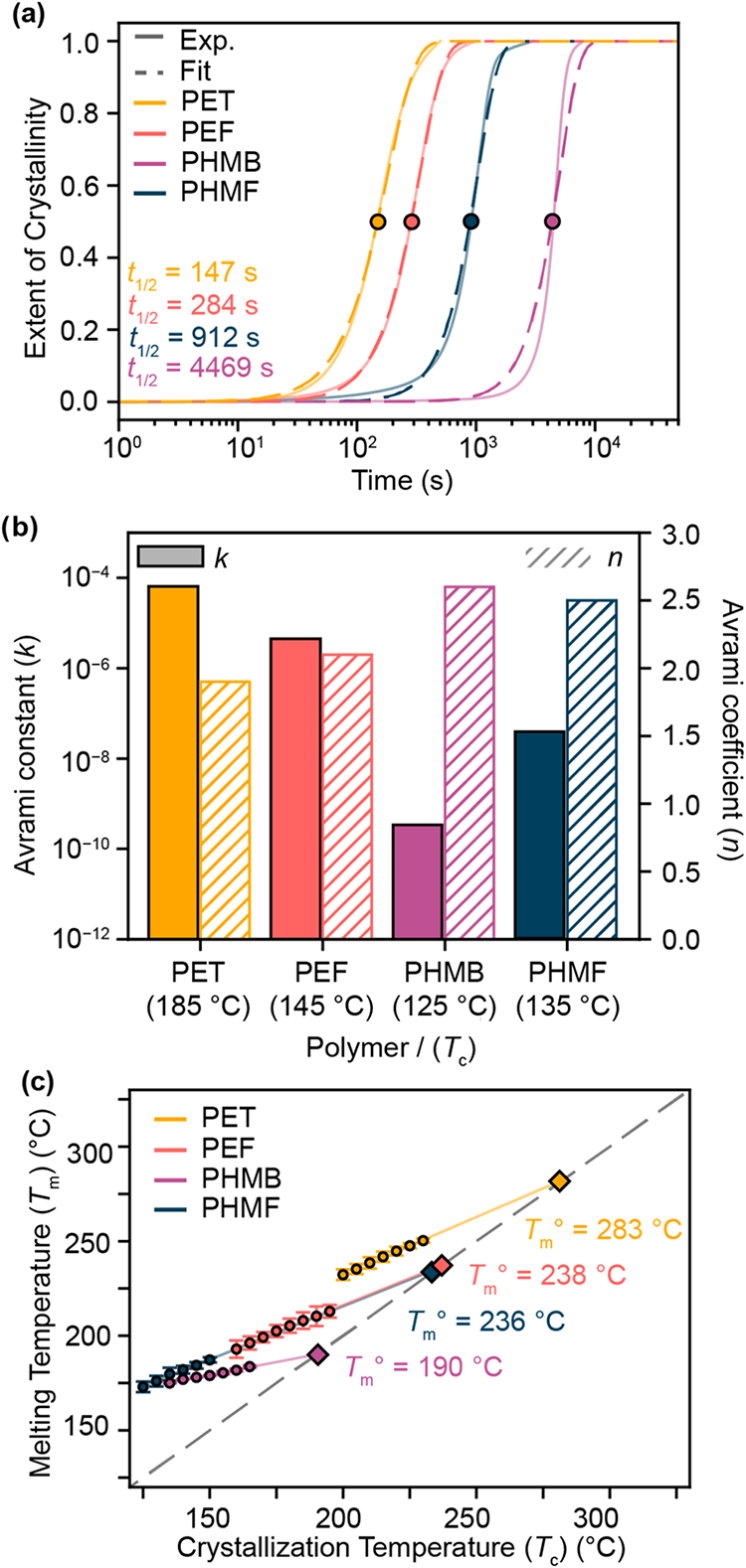
Isothermal crystallization experiments of polyesters: (a) Avrami analysis at intermediate crystallization temperatures (PET: *T*_c_ = 185 °C, PEF: *T*_c_ = 145 °C, PHMB: *T*_c_ = 125 °C, PHMF: *T*_c_ = 135 °C), (b) parameters obtained from fitting crystallization curve (a) to the Avrami equation (eq S1), and (c) Hoffman–Weeks analysis to obtain the *T*_m_°.

Next, the intrinsic thermodynamic stability of the polyester crystallites was examined by measuring *T*_m_ following crystallization at several values of *T*_c_ (Figure S22). The *T*_m_ values correlated linearly with *T*_c_, as predicted by Hoffman–Weeks nucleation theory (Figure 4c).^70^ A linear regression of *T*_m_ vs *T*_c_ allows for the estimation of the equilibrium melting temperature (*T*_m_°), which is the hypothetical melting temperature of a perfect polymer crystal of infinite size. The relative order of *T*_m_° matches that of *T*_m_ for the polyesters examined: PET exhibits the highest *T*_m_° (283 °C) followed by PEF (*T*_m_° = 238 °C), PHMF (*T*_m_° = 236 °C), and PHMB (*T*_m_° = 190 °C).

Equilibrium melting temperature is correlated to the thermodynamic stability of the crystalline phase in the absence of kinetic limitations.^70^ The very small difference in *T*_m_° (2 K) and larger difference in *T*_m_ (25 K) for PEF relative to PHMF imply that the crystalline phase of PHMF is as thermodynamically stable as PEF, but kinetic limitations prevent the formation of comparable crystals under normal conditions, which is consistent with the previous observation that PHMF crystallizes more slowly than PEF. In addition, two endothermic events separated by nearly 20 °C are observed for PHMF, suggesting that PHMF forms multiple crystalline phases when slowly crystallized from the melt. This effect has also been reported for both PET and PEF, though with a smaller temperature separation between the two endothermic events.^71,72^ The colder endothermic event is associated with a less thermodynamically stable but more kinetically favorable crystalline phase.

### Analysis of Polymer Chain Mobility via Solid-State NMR Spectroscopy

Solid-state NMR spectroscopy offers an effective method to quantitatively study chain structure and mobility in solid polymer samples.^73,74^ Variable contact time-cross-polarization-magic angle spinning (VCT-CP-MAS) NMR spectroscopy measuring the relaxation of the ester carbonyl as a function of contact time was used to study polyester rigidity (eq S2 and Figure 5a).^13^ The trend in the time constant for longitudinal rotating frame relaxation (*T*_1ρ_) matches the trend in crystallization half-life—PET has the shortest *T*_1ρ_ of 8.7 ms followed by PEF (*T*_1ρ_ = 17.8 ms), PHMF (*T*_1ρ_ = 35.9 ms), and PHMB (*T*_1ρ_ = 54.7 ms)—reinforcing the hypothesis that the polyesters based on the asymmetric repeat structures have less mobile chain structures than those based on the symmetric repeat structures (Figure 5b). This is further supported by the observation that the time constant of magnetization buildup (*T*_CH_) of PHMF (*T*_CH_ = 1.16 ms) and PHMB (*T*_CH_ = 1.13 ms) is less than those of PET (*T*_CH_ = 1.28 ms) and PEF (*T*_CH_ = 1.39 ms). These data are consistent with the conclusion that PHMF exhibits a remarkably rigid backbone, which may contribute to enhancements in thermal and barrier properties;^13^ however, they fail to elucidate the origin of major property differences between PHMF and PHMB (e.g., *T*_g_).

**Figure 5:**
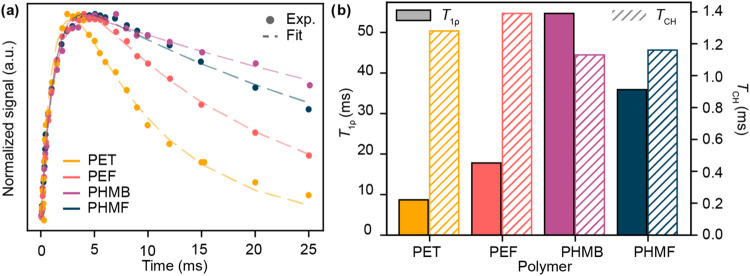
Solid-state NMR spectroscopy of selected polyesters: (a) intensity of carbonyl signal from VCT-CP-MAS NMR spectroscopy of polyesters with fit and (b) extracted *T*_1ρ_ and *T*_CH_ parameters for each polyester.

### Computational Analysis of Polyester Structure–Property Relationships

To better understand the origins of the observed differences in chain mobility and thermal properties, molecular dynamics (MD) simulations were used to estimate polymer properties (Table S7). To assess the validity of the model, the amorphous density was computed for the equilibrated system and compared to available experimental data. The computed densities of 1.29 and 1.38 g/cm^3^ for PET and PEF, respectively, were in reasonable agreement with the experimentally measured amorphous densities for PET and PEF (1.33 and 1.43 g/cm^3^, respectively) (Figure 6a).^13^ The computed densities of PHMB (*d*_comp._ = 1.24 g/cm^3^) and PHMF (*d*_comp._ = 1.36 g/cm^3^) were notably lower than those of the symmetric polyesters. In addition, the furan-based polyesters exhibited higher densities than the phenyl-based polyesters. To determine whether the model recapitulated observed trends in backbone stiffness, rigidity was quantified using the characteristic ratio (*C*_∞_), which compares a polymer’s observed radius of gyration to that of a freely jointed chain.^75^ The computed characteristic ratios of asymmetric polyesters PHMB (*C*_∞_ = 7.2) and PHMF (*C*_∞_ = 6.8) are significantly higher than those of the symmetric polyesters PET (*C*_∞_ = 5.2) and PEF (*C*_∞_ = 4.8), which agrees with our experimental observations, but also reaffirms that the large property differences between PHMF and PHMB cannot exclusively be explained by differences in backbone rigidity (Figure 6b).

**Figure 6:**
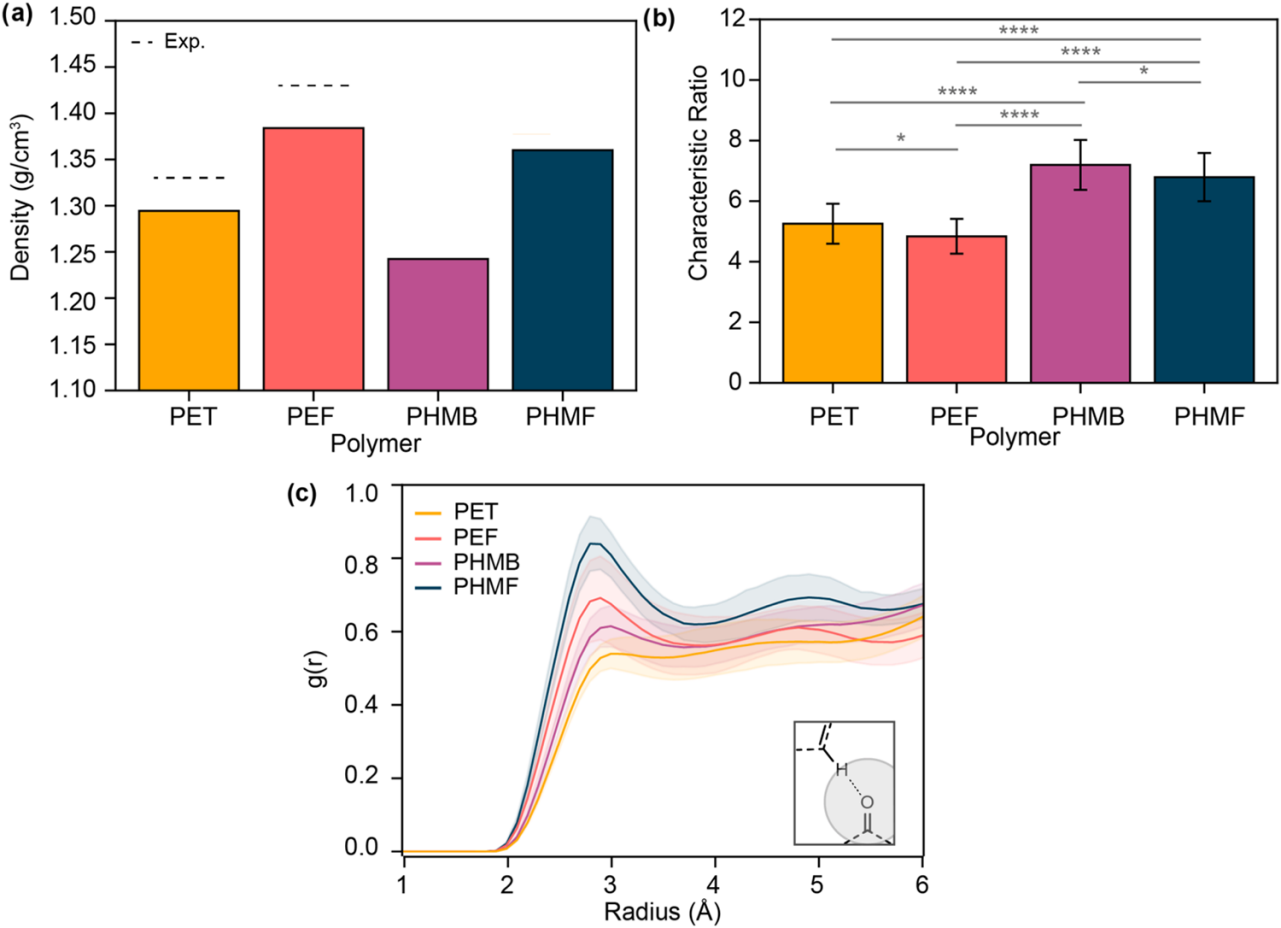
Computed properties from molecular dynamics simulations on polyesters including (a) amorphous densities (compared to experiment), (b) characteristic ratio, and (c) interchain radial distribution function (RDF) between the carbonyl and adjacent aromatic ring. Error bars represent one standard error from the mean in 25 independent simulations. Shaded regions represent one standard error from the mean in 5 independent simulations (*: *p* < 0.05, **: *p* < 0.01, ***: *p* < 0.001, ****: *p* < 0.0001).

Previous work has highlighted how noncovalent intermolecular interactions between furanic protons and carbonyl groups impact chain conformation in the amorphous and crystalline phases.^14^ These interactions were examined by plotting the interchain radial distribution function (RDF) between aromatic protons and carbonyl oxygens for each polyester (Figure 6c). The RDFs suggest that furan-containing polyesters have a significantly higher density of aromatic protons within moderate noncovalent interaction range (<3 Å) of a carbonyl on an adjacent chain relative to their phenyl analogs. This effect is especially pronounced for PHMF, which has a notably higher density of aromatic protons around the carbonyl than does PHMB. Thus, this model supports the hypothesis that noncovalent interactions in furan-based polyesters are significant contributors to enhancements in density and *T*_g_ relative to phenyl-based polyesters. With both its rigid asymmetric geometry and furan structure, PHMF is unique among the polyesters studied in both exhibiting a highly rigid repeat unit and noncovalent interactions induced by the furan ring.

## Conclusions

Furan-derived polyesters sourced from biomass are promising alternatives to petroleum-derived analogs. PHMF has the highest possible furan content for a polyester and provides a new system to probe structure–property relationships beyond the more well-studied FDCA-based polymers. We developed two polycondensation methods to synthesize PHMF from its methyl ester monomer and demonstrated facile chemical recycling. Thermomechanical, spectroscopic, and MD simulation results indicate that PHMF has high chain rigidity and participates in noncovalent interactions, leading to its high computed density and *T*_g_. Together, these results show how exploration of new structural motifs such as asymmetric furan-based monomers adds new possibilities for sustainably sourced performance-advantaged bioplastics.

## Experimental Section

### General Procedure for the Melt-Phase Polycondensation of Hydroxyester

Hydroxyester monomer and (Oct)_2_SnO (0.5 mol%) were added to an oven-dried three-neck flask equipped with a distillation head and overhead stirrer before evacuating and backfilling with nitrogen three times to remove oxygen (Figure S9). The vessel was subsequently heated to 140 °C with stirring for 16 h, 160 °C for 2 h, and 180 °C for 4 h under nitrogen. The reaction vessel was subsequently evacuated (<1 Torr) and stirred for an additional 16 h. At the end of the reaction, the flask was allowed to cool under nitrogen. The crude material was dissolved in TFA/CH_2_Cl_2_ (50% v/v) and precipitated in methanol. Precipitated polymer was filtered and washed with methanol three times before drying under vacuum at 80 °C overnight.

### General Procedure for the Solution-Based Polycondensation of Hydroxyester

Hydroxyester monomer, (Oct)_2_SnO (1 mol%), and toluene (0.1 M) were added to a round-bottom flask equipped with a Dean–Stark trap and reflux condenser before sparging with nitrogen for 15 min. The reaction flask was lowered into an oil bath set to 180 °C and allowed to reflux for 18 h under nitrogen. The vessel was subsequently cooled, and toluene was removed via rotary evaporation. The crude material was dissolved in TFA/CH_2_Cl_2_ (50% v/v) and precipitated in methanol. Precipitated polymer was filtered and washed with methanol three times before drying under vacuum at 80 °C overnight.

### General Procedure for the Postcondensation of PHMF

PHMF and (Oct)_2_SnO (1 mol%) were ground together into a fine powder using a mortar and pestle before transferring to an oven-dried round-bottom flask. The round-bottom flask was evacuated (<1 Torr) and heated in an oil bath set at 190 °C for 16 h. At the end of the reaction, the vessel was backfilled with nitrogen and allowed to cool. The crude material was dissolved in TFA/CH_2_Cl_2_ (50% v/v) and precipitated in methanol. Precipitated polymer was filtered and washed with methanol three times before drying under vacuum at 80 °C overnight.

### Depolymerization of PHMF

PHMF, K_2_CO_3_ (10 mol%), and methanol (0.1 M) were added to an oven-dried round-bottom flask equipped with a reflux condenser. The reaction mixture was lowered into an oil bath set at 80 °C and allowed to reflux for 1 h. The vessel was then cooled before it was concentrated via rotary evaporation. The crude mixture was partitioned in a separatory funnel between ethyl acetate and water. The organic layer was extracted three times, dried with MgSO_4_, concentrated with rotary evaporation, and purifyied by column chromatography. Product fractions were concentrated via rotary evaporation and dried overnight under vacuum.

### Isothermal Crystallization of Polyesters

Isothermal crystallization measurements were performed using a TA Instruments DSC 2500. Polymer samples (5 mg) were heated to *T*_m_ + 30 °C at 10 °C/min, held at *T*_m_ + 30 °C for 5 min, cooled to the desired *T*_c_ at 200 °C/min, and held at *T*_c_ until crystallization was complete (see SI). Each sample was cycled at seven different values of *T*_c_. Time, temperature, and heat flow data were exported from TA Trios software and analyzed using Python.

### Variable Contact Time Solid-State NMR

Polymer samples were prepared by rapidly cooling a melted polymer film to minimize crystallinity. Films were pulverized into powders via cryomilling and dried for over 1 week to remove moisture before packing into rotors. Rotors were spun at the magic angle with a spinning speed of 7 kHz and a temperature of 35 °C. Cross-polarization (CP) measurements were performed with a 2.7 μs ^1^H excitation pulse followed by a 2 ms contact time step (see SI). For variable contact time measurements, the length of the contact time step was varied between 0.01 and 25 ms. Spectral processing was performed with MestReNova and Python.

### Computational Methods

MD simulations were designed using the Schrödinger platform Maestro Materials Science 5.1.125. Homopolymer amorphous cells were constructed using the Maestro Materials Science Polymer Builder function to contain 10 polymer chains, each consisting of 100 repeat units. Amorphous polymer cells were equilibrated using a multistep annealing procedure adapted from previous reports.^76,77^ Polyester properties were computed from an average of 25 independent cell simulations at 298 K unless otherwise stated. Cell density was extracted from the final 1 ns of the simulation. The Maestro Materials Science Polymer Chain Analysis tool was used to calculate end-to-end chain distance and segment length in the polymer melt (600 K), which were used to calculate the characteristic ratio (eq S3). Radial distribution functions were computed using the Maestro Materials Radial Distribution Function tool in five independent simulations.^78–80^

## Supplementary Material



## Notes

Additional experimental details, material characterization (NMR, DSC, TGA, MALDI-TOF, GPC, DMA), photographs, synthesis optimization data, and supporting equations (PDF)

## Abbreviations

PHMF: poly­(5-hydroxymethyl furanoate)
PEF: poly­(ethylene furanoate)
PET: poly­(ethylene terephthalate)
PHMB: poly­(*m*-hydroxymethyl benzoate)
FDCA: furan-2,5-dicarboxylic acid
*T*_g_: glass transition temperature
FA: furoic acid
HMF: 5-hydroxymethyl furfural
MHMF: methyl 5-hydroxymethyl furanoate
HMFA: 5-hydroxymethyl furoic acid
DP: average degree of polymerization
DSC: differential scanning calorimetry
NMR: nuclear magnetic resonance spectroscopy
HSQC: heteronuclear single quantum coherence NMR spectroscopy
HMBC: heteronuclear multiple bond correlation nuclear magnetic resonance spectroscopy
COSY: correlated NMR spectroscopy
MALDI-TOF: matrix-assisted laser desorption ionization time-of-flight mass spectrometry
GPC: gel permeation chromatography
*M*_n,GPC_: number-average molar mass measured by GPC
*Đ*: dispersity
TFA: trifluoroacetic acid
DMA: dynamic mechanical analysis
*T*_c_: crystallization temperature
*Q*_c_: heat of crystallization
*t*_1/2_: half-life of crystallization
*n*: Avrami exponent
*k*: Avrami rate constant
*T*_m_°: equilibrium melting temperature
VCT-CP-MAS: variable contact time-cross-polarization-magic angle spinning
*T*_CH_: time constant of magnetization build up
*T*_1ρ_: time constant for longitudinal rotating frame relaxation
MD: molecular dynamics
*d*_comp_: computed density
*C*_∞_: characteristic ratio
RDF: radial distribution function
