# Supplementary material for: Enhancing Biopolyester Backbone Rigidity with an Asymmetric Furanic Monomer
Source: ACS Sustain Chem Eng. 2025 Oct 8;13(41):17625–34. doi: 10.1021/acssuschemeng.5c07913 (PMC12541805; doi:10.1021/acssuschemeng.5c07913)
Supplement: Supplementary file 1 [file sc5c07913_si_001.pdf]

Supporting Information:

Enhancing bio-polyester backbone rigidity with an asymmetric furanic monomer

Cristian P. Woroch,[1,2] Bennett Addison,[2] Alexandra Stovall,[2] Erik Rognerud,[2] Clarissa Lincoln,[2] Joel Miscall,[2] Gloria Rosetto,[2] Matthew W. Kanan[1], Nicholas A. Rorrer,[2,*] Gregg T. Beckham[2,*]

[1]*Department of Chemistry, Stanford University, 337 Campus Drive, Stanford, California, 94305, United States*

[2] *Renewable Resources and Enabling Sciences Center, National Renewable Energy Laboratory, Golden, CO 80401, USA.*

* correspondence: nicholas.rorrer@nrel.gov; gregg.beckham@nrel.gov

Number of pages: 36

Number of figures: 22

Number of tables: 7

Table of Contents **Page**

1. Experimental methods S1-8
2. Supporting figures S9-28
3. Supporting tables S29-32
4. Supporting equations S33-34
5. Supporting references S35-36

1. Experimental methods

1.1. Chemicals and materials

5-hydroxymethyl furoic acid (HMFA), dimethyl terephthalate (DMT), and dimethyl furan-2,5-dicarboxylate (MFDC) were purchased from Aaron Chemicals LLC. *m*-hydroxymethyl benzoic acid (*m*-HMBA), and dioctyl tin oxide ($(Oct)_2SnO$) were purchased from AA Blocks LLC. Except for polyester monomers, all chemicals were used without additional purification. Polyester monomers were recrystallized twice and dried before usage (see below).

1.2. General methods

NMR spectra were obtained at 23 °C on either a 600, 500, or 400 MHz Varian Unity Inova spectrometer or a 400 MHz Bruker spectrometer. Deuterated solvents were used as received from Cambridge Isotopes. Differential scanning calorimetry (DSC) was performed on a TA Instruments Discovery Differential Scanning Calorimeter (DSC) 2500 at the Stanford Soft Materials Facility. Samples of ~5 mg were sealed in standard aluminum “Tzero” pans with lids, purchased from DSC Consumables. Heat flow to the sample in mW was recorded relative to a reference pan and lid prepared without sample. Measurements were performed under 50 mL/min dry N_2 flow with a temperature ramp rate of 10 °C/min from 50 °C to 700 °C. Glass transition temperatures (T_g) were determined by taking the midpoint of the transition curve on the second heating cycle. Thermogravimetric analysis (TGA) was performed on a TA Instruments 5500 at the Stanford Soft Materials Facility. Samples of ~10 mg were loaded onto 100 µL alumina crucibles and heated to desired temperature under N_2 flow at a rate of 10 °C/min. Powder X-ray diffraction was performed under ambient conditions on a Bruker D8 Advance diffractometer equipped with a Cu anode ($K_{\alpha 1}$ = 1.54060 Å, $K_{\alpha 2}$ = 1.54443 Å, $K_{\alpha 2}/K_{\alpha 1}$ = 0.5). Matrix-Assisted Laser Desorption Ionization Time of Flight Spectrometry (MALDI) was performed at the Stanford Peptide and Nucleic Acid Facility (Stanford, CA). Polymer samples were dissolved in THF (~5 mg/mL). The MALDI matrix was prepared using sinapinic acid (~10 mg/mL) in 1:1 acetonitrile/water with 1 wt% TFA. Each MALDI sample was prepared by combining 2 µL of the MALDI matrix with 1.5 µL of the polymer solution and depositing them onto a MALDI plate. The solution was dried before analysis with an Applied Biosystems Voyager DE RP MALDI-TOF Spectrometer (Laser energy ~2.5 µJ). Gel permeation chromatography (GPC) was conducted using an Agilent 1260 Infinity II LC system, including an isocratic pump, vial sampler, and multicolumn thermostat. The eluent was separated over three sequential Agilent PL HFIPgel 250 x 4.6 mm columns with a preceding guard column. Hexafluoroisopropanol (HFIP) with 20 mM NaTFA was used as the mobile phase. Samples were prepared at a concentration of 5 mg/mL and filtered through a PTFE syringe filter. Measurements were conducted at a flow rate of 0.35 mL/min, column oven temperature of 40 °C, and with a 100 uL sample injection volume. Number average molar mass ($M_{n,GPC}$), weight average molar mass

($M_{w,GPC}$), and dispersity ($Đ$) were determined using a miniDawn TREOS multi-angle light scattering (MALS) detector (Wyatt Technology) in combination with an Optilab T-rEX differential refractive index (dRI) detector (Wyatt Technology). Wyatt Technologies Astra Software was used for data analysis. Solid-state NMR spectroscopy (ssNMR) was performed on a 200 MHz (4.7 Tesla) Bruker AvanceIII HD NMR spectrometer equipped with a Bruker 4-mm HX probe.

1.3. Synthesis of methyl 5-hydroxymethyl furanoate (MHMF)

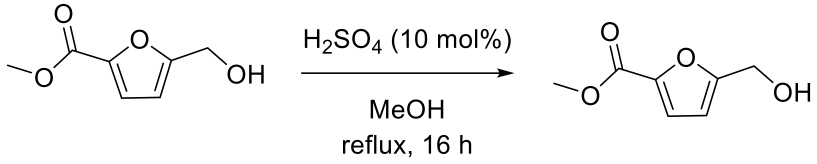

Methyl 5-hydroxymethyl furanoate (MHMF) was synthesized by adapting a previously reported method.[81] 5-hydroxymethyl furoic acid (HMFA) (1 equiv) and methanol (0.5 M) were added to an oven-dried round bottom flask with a stir bar. Reaction was mixed until HMFA was completely dissolved. Concentrated sulfuric acid (H_2SO_4) (0.1 equiv) was added to the flask. Flask was equipped with a reflux condenser and lowered into a silicone oil bath set at 80 °C. Reaction was allowed to stir under reflux overnight. Reaction completion was monitored by TLC (eluent = 1:1 hexane:ethyl acetate, $r_f \approx 0.5$). At the end of the reaction, flask was allowed to cool. Saturated sodium bicarbonate was added to neutralize the reaction as measured by pH test strips. Solution was concentrated via rotoevaporation before partition in a separatory funnel between ethyl acetate and water. Organic layer was extracted three times before drying over $MgSO_4$, concentrated with rotary evaporation, and purified by column chromatography (eluent = 0 to 100% ethyl acetate in hexanes, 20 minutes). Product fractions were concentrated via rotary evaporation and dried overnight under high vacuum. Crude oil product was subsequently diluted with cyclopentyl methyl ether (CPME) or diethyl ether (Et_2O) (~100 v/v%) and cooled in a freezer (−18 °C) overnight to yield a white crystalline solid. Mother liquid was decanted and the resulting crystals were dried overnight under high vacuum yielding a white solid (10g scale, 85% yield). ^{1}H NMR ($CDCl_3$, 400 MHz, δ): 7.12 (1H, d, J = 3.4 Hz), 6.41 (1H, d, J = 3.4 Hz), 4.67 (2H, s), 3.89 (3H, s). ^{13}C NMR ($CDCl_3$, 101 MHz, δ): 159.18, 158.32, 144.08, 118.89, 109.49, 57.59, 51.99.

1.4. Synthesis of methyl *m*-hydroxymethyl benzoate (MHMB)

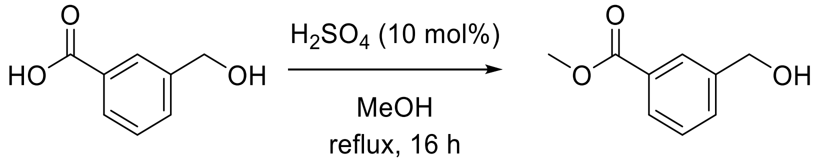

Methyl *m*-hydroxymethyl benzoate (MHMB) was synthesized by a similar method to MHMF. *M*-hydroxymethyl benzoic acid (HMFA) (1 equiv) and methanol (0.5 M) were added to an oven-dried round bottom flask with a stir bar. Reaction was mixed until HMFA was completely dissolved. Concentrated sulfuric acid (H_2SO_4) (0.1 equiv) was added to the flask. Flask was

equipped with a reflux condenser and lowered into a silicone oil bath set at 80 °C. Reaction was allowed to stir under reflux overnight. Reaction completion was monitored by TLC (eluent = 1:1 hexane:ethyl acetate, $r_f \approx 0.4$). At the end of the reaction, flask was allowed to cool. Saturated sodium bicarbonate was added to neutralize the reaction as measured by pH test strips. Solution was concentrated via rotoevaporation before it was partitioned in a separatory funnel between ethyl acetate and water. Organic layer was extracted three times before drying with $MgSO_4$, concentrated with rotary evaporation, and purified by column chromatography (eluent = 0 to 100% ethyl acetate in hexanes, 20 minutes). Product fractions were concentrated via rotary evaporation and dried overnight under high vacuum. Clear oil product could not be recrystallized and was therefore used without further purification (10g scale, ~99% yield). ^{1}H NMR ($CDCl_3$, 400MHz, δ): 8.04 (1H, s), 7.98 (1H, d, J = 7.6 Hz), 7.52 (1H, d, J = 7.5 Hz), 7.38 (1H, t, J = 7.7 Hz), 4.69 (2H, s), 3.88 (3H, s). ^{13}C NMR ($CDCl_3$, 101 MHz, δ): 167.16, 141.37, 131.43, 128.68, 128.58, 127.91, 64.55, 52.18.

1.5. Synthesis of poly(5-hydroxymethyl furanoate) (PHMF) – melt phase

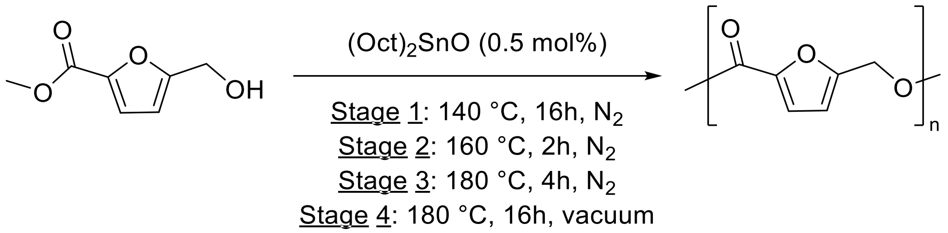

Recrystallized MHMF (1 equiv) and dioctyl tin oxide ($(Oct)_2SnO$) (0.5 mol%) were added to an oven-dried three-neck flask equipped with a distillation head and overhead stirrer (Figure S9). Flask was flushed and evacuated with nitrogen three times to remove oxygen. While stirring under nitrogen, the reaction vessel was heated to 140 °C and held at that temperature overnight, resulting in the formation of a white solid. The reaction vessel was heated to 160 °C for 2 hours, and subsequently, to 180 °C for 4 hours. The reaction vessel was subsequently evacuated and stirred for an additional 16 hours. At the end of the reaction, the flask was allowed to cool under nitrogen. The crude material was dissolved in TFA/CH_2Cl_2 (50 %v/v) and precipitated in methanol. Precipitated polymer was filtered and washed with methanol thrice before drying under vacuum at 70 °C overnight to yield an off-white solid of poly(5-hydroxymethyl furanoate) (PHMF). (5g scale, 73% yield, $M_{n,GPC}$ = 13.7 kDa, $Đ$ = 2.3, dn/dc = 0.253, T_g = 77 °C). ^{1}H NMR ($CDCl_3$/TFA 1:1, 400 MHz, δ): 7.33 (1H, d, J = 3.6 Hz), 6.72 (1H, d, J = 3.6 Hz), 5.41 (2H, s). ^{13}C NMR ($CDCl_3$/TFA 1:1, 101 MHz, δ): 160.88, 153.83, 143.19, 121.33, 113.68, 58.96.

1.6. Synthesis of poly(5-hydroxymethyl furanoate) (PHMF) – solution phase

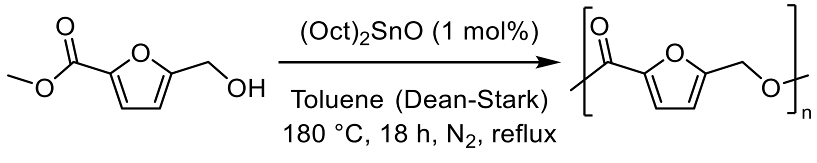

Recrystallized MHMF (1 equiv), dioctyl tin oxide ($(Oct)_2SnO$) (1 mol%), and toluene (0.1 M) were added to a round bottom flask containing a stir bar and equipped with a Dean-Stark trap,

reflux condenser, and an inlet and outlet to allow for continuous flow of N_2. Reaction flask was lowered into an oil bath set to 180 °C and allowed to reflux for 18 hours. Over the course of the reaction, inhomogeneous polymer particles were formed and became suspended in solution or adhered to the reaction flask. At the end of the allotted reaction time, the toluene was removed via rotary evaporation. The crude material was dissolved in TFA/CH_2Cl_2 (50 %v/v) and precipitated in methanol. Precipitated polymer was filtered and washed with methanol thrice before drying under vacuum at 70 °C overnight to yield a white solid of poly(5-hydroxymethyl furanoate) (PHMF). (2 g scale, 96% yield, $M_{n,GPC}$ = 10.3 kDa, $Đ$ = 1.7, dn/dc = 0.261, T_g = 77 °C).

1.7. Post-polymerization solid state polycondensation of PHMF

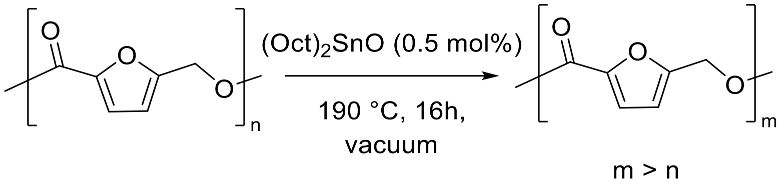

PHMF and (Oct)$_2$SnO (1 mol%) were ground together into a fine powder using a mortar and pestle before transferring to an oven-dried round bottom flask. Round bottom flask was evacuated and heated in an oil bath set at 190 °C for 16 hours. At the end of the reaction, the flask was allowed to cool under nitrogen. The crude material was dissolved in TFA/CH_2Cl_2 (50 %v/v) and precipitated in methanol. Precipitated polymer was filtered and washed with methanol thrice before drying under vacuum at 70 °C overnight to yield an off-white solid. (1g scale, 94% yield, $M_{n,GPC}$ = 48.8 kDa, $Đ$ = 1.1, dn/dc = 0.259, T_g = 83 °C).

1.8. Depolymerization of PHMF

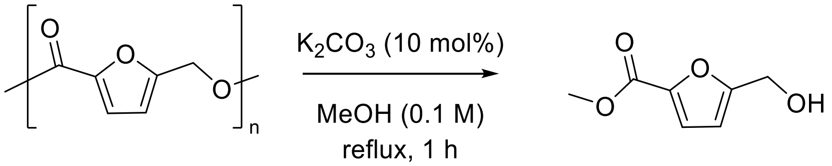

PHMF (1 equiv), potassium carbonate (K_2CO_3) (10 mol%), and methanol (0.1 M) were added to an oven-dried round bottom flask with a stir bar and reflux condenser. The reaction mixture was lowered into an oil bath set at 80 °C and allowed to reflux for one hour. At the end of the reaction, the solution was allowed to cool. Solution was concentrated via rotary evaporation before partition in a separatory funnel between ethyl acetate and water. Organic layer was extracted three times before drying with $MgSO_4$, concentrating with rotary evaporation, purifying by column chromatography (eluent = 0 to 100% ethyl acetate in hexanes, 20 minutes). Product fractions were concentrated via rotary evaporation and dried overnight under high vacuum to yield a clear oil. (1g scale, 85% yield, without recrystallization).

1.9. Synthesis of poly(*m*-hydroxymethyl benzoate) (PHMB) – melt phase

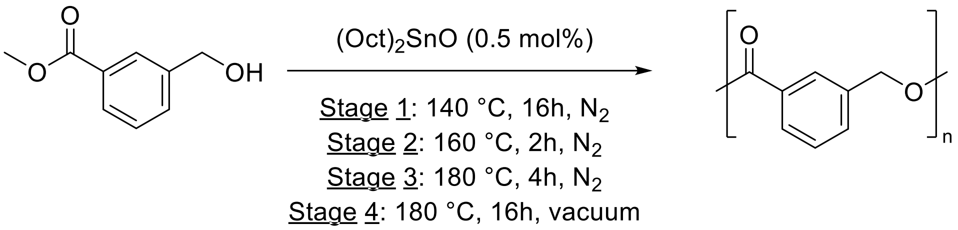

Poly(*m*-hydroxymethyl benzoate) (PHMB) was synthesized using the same method described for PHMF. MHMB (1 equiv) and dioctyl tin oxide ($(Oct)_2SnO$) (0.5 mol%) were added to an oven-dried three-neck flask equipped with a distillation head and overhead stirrer (Figure S9). Flask was flushed and evacuated with nitrogen three times to remove oxygen. While stirring under nitrogen, the reaction vessel was heated to 140 °C and held at that temperature overnight, resulting in the formation of a white solid. The reaction vessel was heated to 160 °C for 2 hours, and subsequently, to 180 °C for 4 hours. The reaction vessel was subsequently evacuated and stirred for an additional 16 hours. At the end of the reaction, the flask was allowed to cool under nitrogen. The crude material was dissolved in TFA/CH_2Cl_2 (50 %v/v) and precipitated in methanol. Precipitated polymer was filtered and washed with methanol thrice before drying under vacuum at 70 °C overnight to yield a white solid. (5g scale, 90% yield, $M_{n,GPC}$ = 7.0 kDa, $Đ$ = 2.7, dn/dc = 0.273, T_g = 63 °C). ^{1}H NMR ($CDCl_3$/TFA 1:1, 400MHz, δ): 8.19 (1H, broad s), 8.10 (1H, broad s), 7.78 (1H, broad s), 7.56 (1H, broad s) 5.51 (2H, s). ^{13}C NMR ($CDCl_3$/TFA 1:1, 100MHz, δ): 169.06, 135.64, 134.04, 130.18, 129.67, 129.28, 129.15, 67.51.

1.6. Synthesis of poly(*m*-hydroxymethyl benzoate) (PHMB) – solution phase

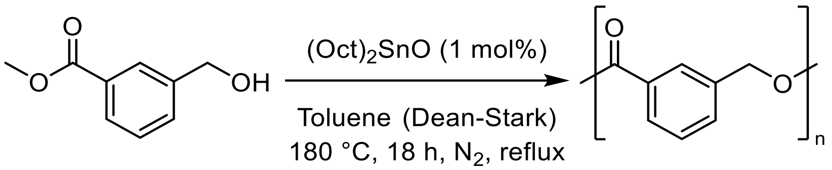

MHMB (1 equiv), dioctyl tin oxide ($(Oct)_2SnO$) (1 mol%), and toluene (0.1 M) were added to a round bottom flask containing a stir bar and equipped with a Dean-Stark trap, reflux condenser, and an inlet and outlet to allow for continuous flow of N_2. Reaction flask was lowered into an oil bath set to 180 °C and allowed to reflux for 18 hours. Over the course of the reaction, inhomogeneous polymer particles were formed and became suspended in solution or adhered to the reaction flask. At the end of the allotted reaction time, the toluene was removed via rotary evaporation. The crude material was dissolved in TFA/CH_2Cl_2 (50 %v/v) and precipitated in methanol. Precipitated polymer was filtered and washed with methanol thrice before drying under vacuum at 70 °C overnight to yield a white solid of poly(m-hydroxymethyl benzoate) (PHMB). (2 g scale, 69% yield, $M_{n,GPC}$ = 9.9 kDa, $Đ$ = 2.4, dn/dc = 0.262, T_g = 67 °C).

1.10. Synthesis of poly(ethylene furanoate) (PEF)

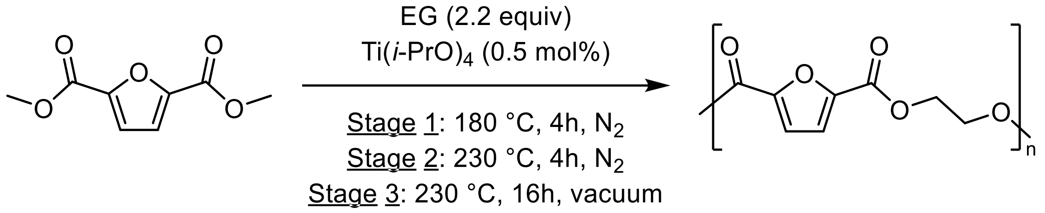

Synthesis of poly(ethylene furanoate) (PEF) was adapted from a previously reported method.[12] Recrystallized dimethyl furan-2,5-dicarboxylate (MFDC) (1 equiv), ethylene glycol (2.2 equiv), and Ti(*i*-PrO)$_4$ (0.5 mol%) were added to an oven-dried three-neck flask equipped with a distillation head and overhead stirrer (Figure S9). Flask was flushed and evacuated with nitrogen three times to remove oxygen. While stirring under nitrogen, the reaction vessel was heated to 180 °C for 4 hours, and subsequently, to 230 °C for 4 hours. The reaction vessel was subsequently evacuated and stirred for an additional 16 hours. At the end of the reaction, the flask was allowed to cool under nitrogen. The crude material was dissolved in TFA/CH_2Cl_2 (50 %v/v) and precipitated in methanol. Precipitated polymer was filtered and washed with methanol thrice before drying under vacuum at 70 °C overnight to yield an off-white solid. (7g scale, 99% yield, $M_{n,GPC}$ = 15.7 kDa, $Đ$ = 3.4, dn/dc = 0.227, T_g = 80 °C). ^{1}H NMR ($CDCl_3$/TFA 1:1, 400 MHz, δ): 7.38 (2H, s), 4.78 (2H, s).

1.11. Synthesis of poly(ethylene terephthalate) (PET)

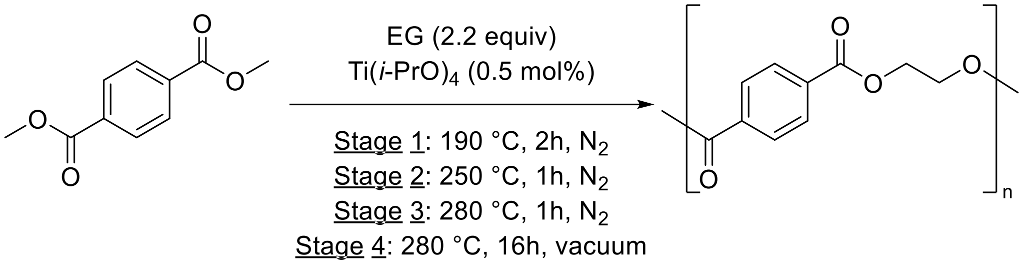

Synthesis of poly(ethylene terephthalate) (PET) was adapted from a previously reported method.[44] Recrystallized dimethyl terephthalate (MFDC) (1 equiv), ethylene glycol (2.2 equiv), and Ti(*i*-PrO)$_4$ (0.5 mol%) were added to an oven-dried three-neck flask equipped with a distillation head and overhead stirrer (Figure S9). Flask was flushed and evacuated with nitrogen three times to remove oxygen. While stirring under nitrogen, the reaction vessel was heated to 190 °C for 2 hours, followed by 250 °C for 1 hour, and subsequently, to 280 °C for 1 hours. The reaction vessel was subsequently evacuated and stirred for an additional 16 hours. At the end of the reaction, the flask was allowed to cool under nitrogen. The crude material was dissolved in TFA/CH_2Cl_2 (50 %v/v) and precipitated in methanol. Precipitated polymer was filtered and washed with methanol thrice before drying under vacuum at 70 °C overnight to yield an off-white solid. (10g scale, 99% yield, $M_{n,GPC}$ = 21.7 kDa, $Đ$ = 2.4, dn/dc = 0.257, T_g = 74 °C). ^{1}H NMR ($CDCl_3$/TFA 1:1, 400 MHz, δ): 7.26 (4H, s), 4.84 (2H, s).

1.12. Determination of monomer purity by differential scanning calorimetry

Determination of monomer purity was performed using a TA Instruments Discovery DSC 2500. Samples of ~5 mg were sealed in standard aluminum "Tzero" pans with lids, purchased from DSC Consumables. Purity determination measurements were performed by first cooling the sample to −20 °C before heating at a rate of 0.5 °C/min until the temperature exceeded 20 °C of the melting endotherm. The purity calculation was performed using the Discovery DSC crystalline purity analysis module which calculates crystalline purity based on van't Hoff analysis.[43]

1.13. Isothermal crystallization of polyesters

Isothermal crystallization measurements of polyesters were performed using a TA Instruments Discovery DSC 2500. Samples of ~5 mg were sealed in standard aluminum "Tzero" pans with lids, purchased from DSC Consumables. Isothermal crystallization measurements were performed using a series of heating and cooling cycles. In each cycle, the polymer was heated at a ramp rate of 10 °C/min to T_m + 30 °C, held at T_m + 30 °C for 5 minutes, then rapidly cooled at 200 °C/min to the desired crystallization temperature T_c, where it was held for a specified crystallization time t_c before the cycle was repeated. The melting temperature, crystallization temperature, and crystallization time were as follows:

Polymer	T_m + 30 (°C)	T_c (°C)	t_c (s)
PET	270	185	1000
PEF	245	145	70,000
PHMB	220	125	170,000
PHMF	220	135	110,000

A total of seven cycles were performed at different values of T_c. Time, temperature, and heat flow data was exported from TA Trios software and analyzed using Python. Each measurement was performed in triplicate.

1.14. Variable contact time solid state NMR spectroscopy

Polymer films were prepared for analysis via melt pressing each sample before rapidly cooling in an ice bath to minimize polymer crystallinity. Films were pulverized into powders via cryomilling and incubated at 55 °C under house vacuum over drierite for one week. Powders were packed into 80-µl Zirconium solids rotors with a polychlorotrifluoroethylene (PCTFE, KelF) drive tip. Rotors were spun at the magic angle at spinning speed of 7 kHz. The temperature at the probe was elevated to 35 °C using a standard probe heater. For CP-MAS, initial ^{1}H polarization was achieved with a 2.7-µs ^{1}H excitation pulse followed by a 2-ms CP step where the ^{13}C channel was set to 50 kHz and the ^{1}H channel was matched to the +1 spinning sideband of the Hartman-Hahn profile using a 10% ramped spin-lockpulse. 1024 scan averages were collected with a 5-s recycle delay, with 80-kHz 1H TPPM decoupling during the 20-ms acquisition time. For Variable Contact Time (VCT) measurements, the same CP conditions were used but the contact pulse was varied in 32 steps from 0.01 to 25 ms, with 256 scan averages were collected for each spectrum. Basic spectral processing was performed using MestReNova version 14. Intensity and contact time were

fit to extract T_{CH} and $T_{1\rho}$ values using python using the SciPy package. $T_{1\rho}$ values were only extracted for non-protonated carbons.

1.15. Computational Methods

Molecular dynamics simulations were designed using the Schrödinger platform Maestro Materials Science 5.1.125 based on Maestro Core 13.7.125. Homopolymer amorphous cells were constructed using the Maestro Materials Science Polymer Builder function. Each polymer cell was constructed with 10 polymer chains each containing 100 repeat units. Amorphous polymer cells were equilibrated using a multistep annealing procedure adapted from previous reports (**Table S6**).[75,76] After equilibration, energy and density were examined to ensure the cell had fully equilibrated. Properties were computed from an average of 25 independent cell simulations at 298 K unless otherwise stated. Cell density was extracted from the final 1 ns of the simulation. The Maestro Materials Science Polymer Chain Analysis tool was used to calculate extended chain length and segment length in the polymer melt (600K) which were used to calculate the characteristic ratio (**Eq. S5**). Radial distribution functions were computed using the Maestro Materials Radial Distribution Function tool in five independent trials.

2. Supporting figures

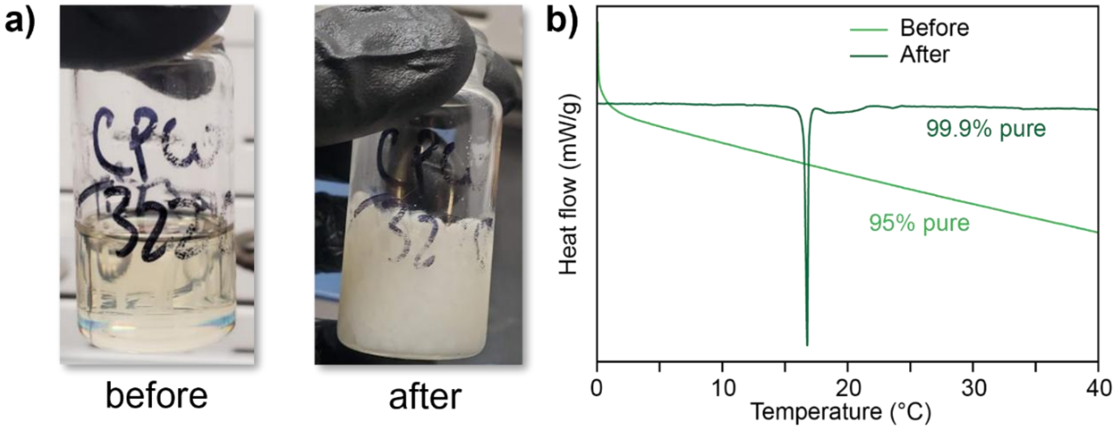

Figure S1. Recrystallization of MHMF. a) before and after photos of MHMF recrystallized in cyclopentyl methyl ether. b) DSC traces with labeled purities determined by NMR spectroscopy and DSC.

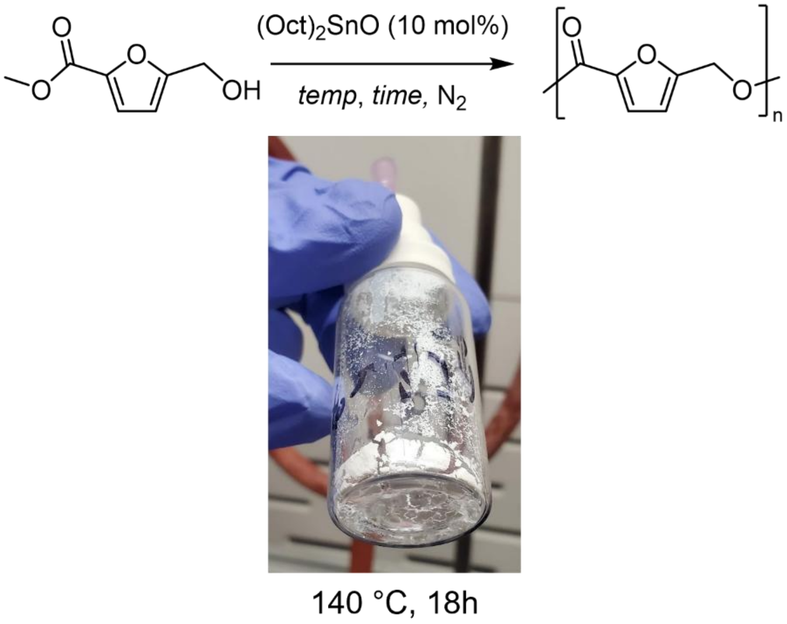

Figure S2. Photo of a small-scale polymerization of MHMF at 140 °C highlighting minimal discoloration.

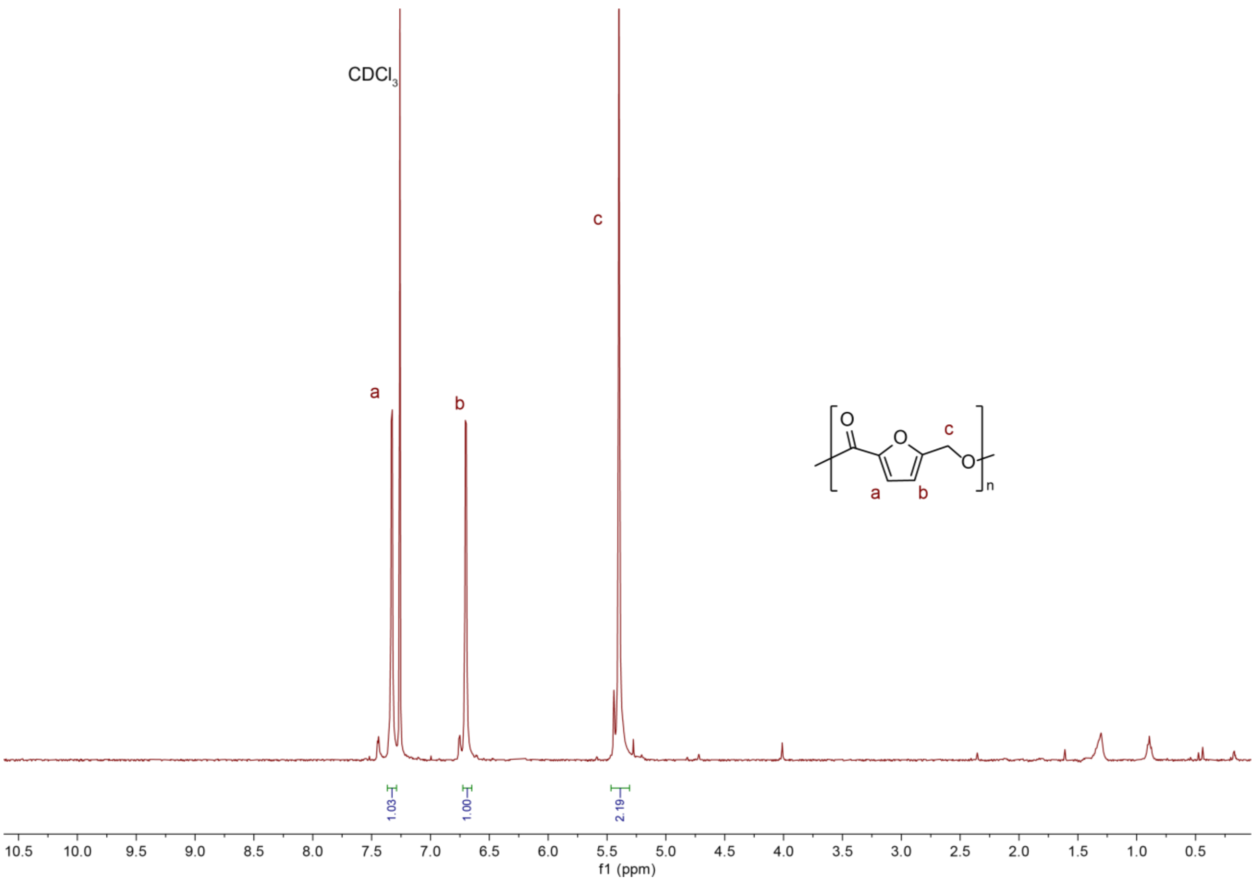

Figure S3. ^{1}H NMR spectrum of PHMF (TFA/$CDCl_3$, 400 MHz).

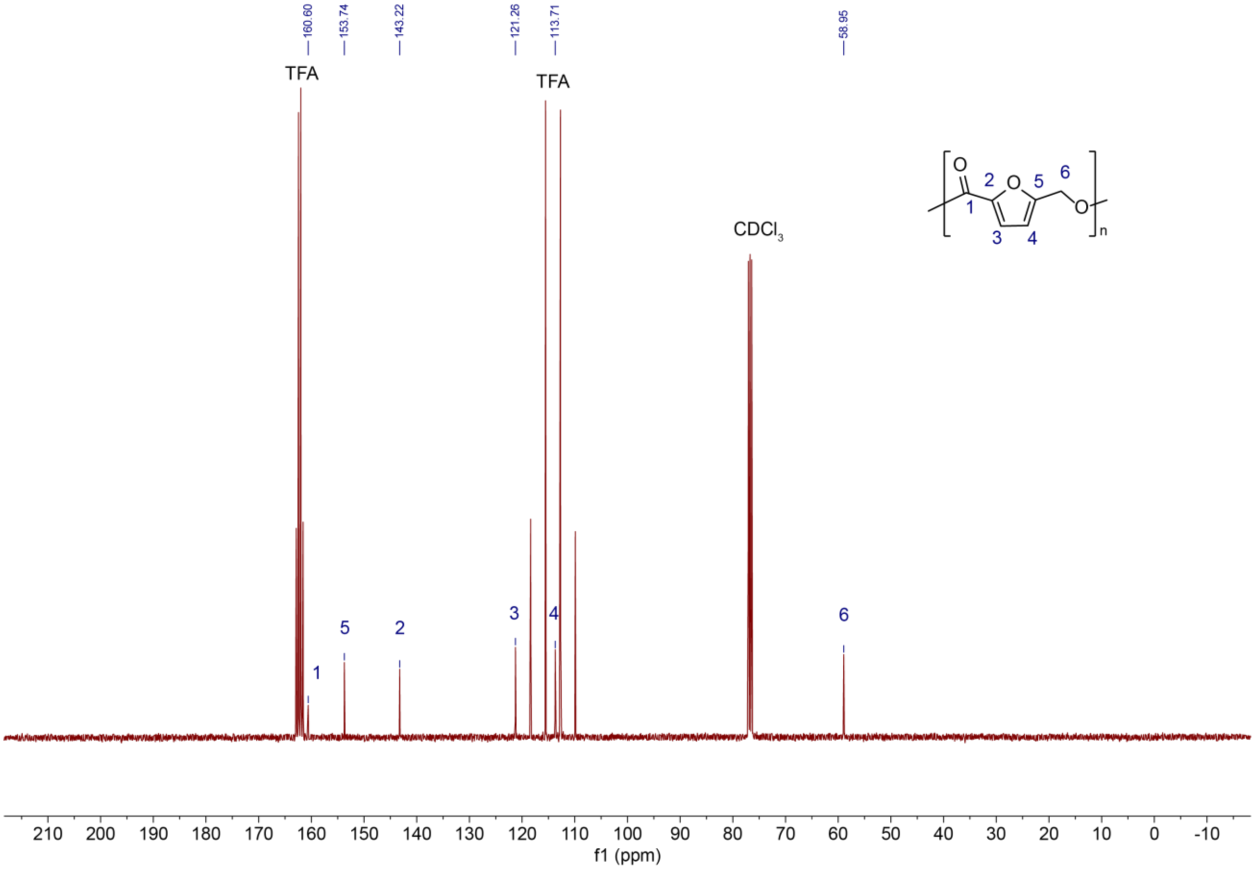

Figure S4. ^{13}C NMR spectrum of PHMF (TFA/$CDCl_3$, 101 MHz).

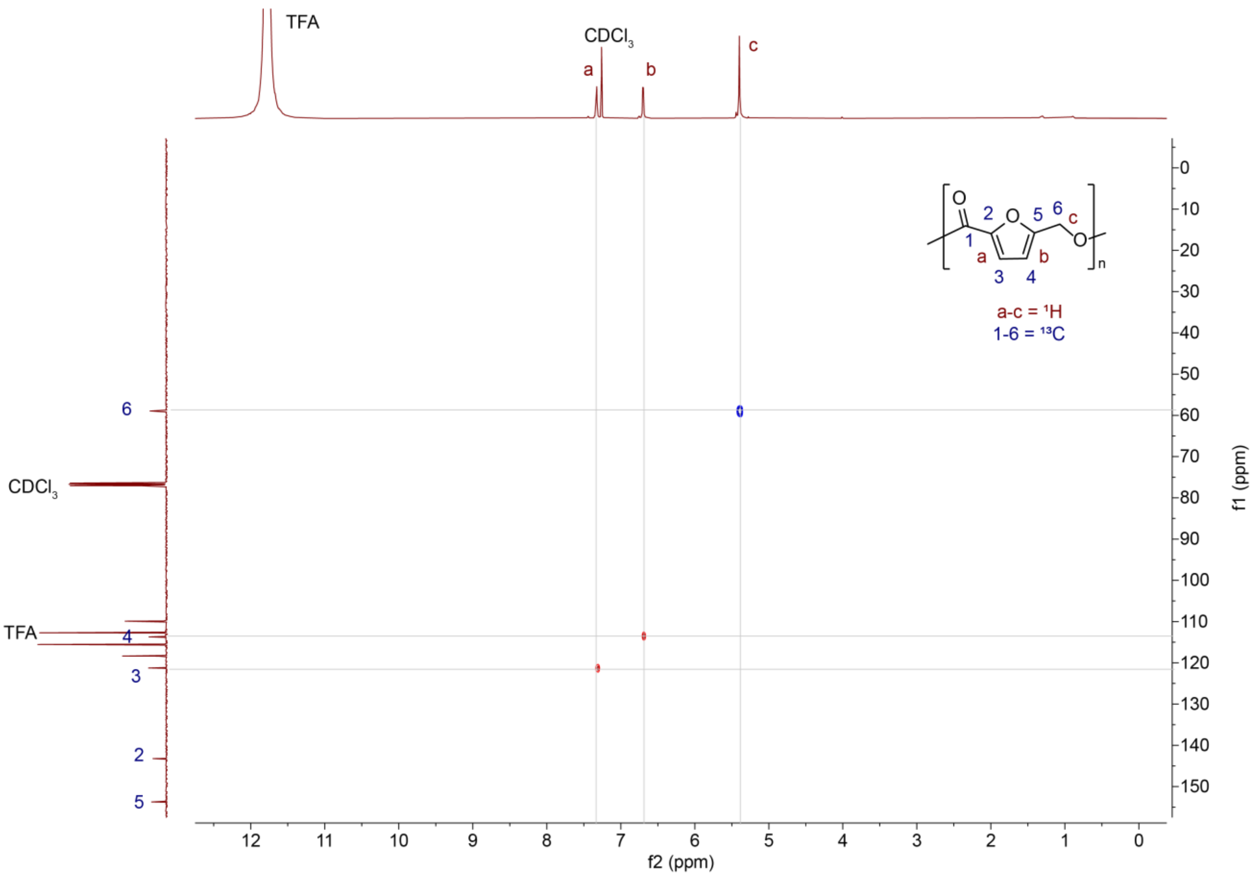

Figure S5. HSQC NMR spectrum of PHMF (TFA/$CDCl_3$, ^{1}H: 400 MHz, ^{13}C: 101 MHz).

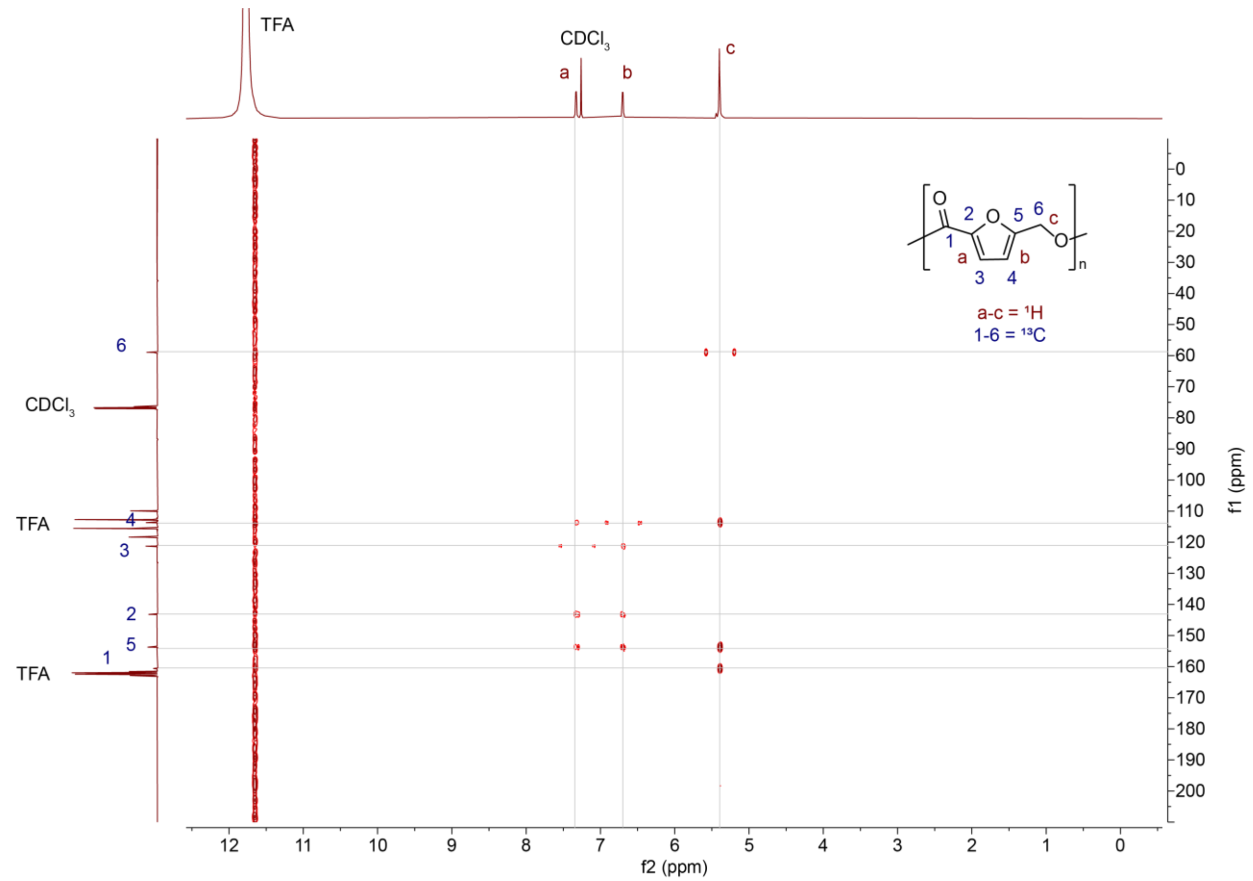

Figure S6. HMBC NMR spectrum of PHMF (TFA/$CDCl_3$, ^{1}H: 400 MHz, ^{13}C: 101 MHz).

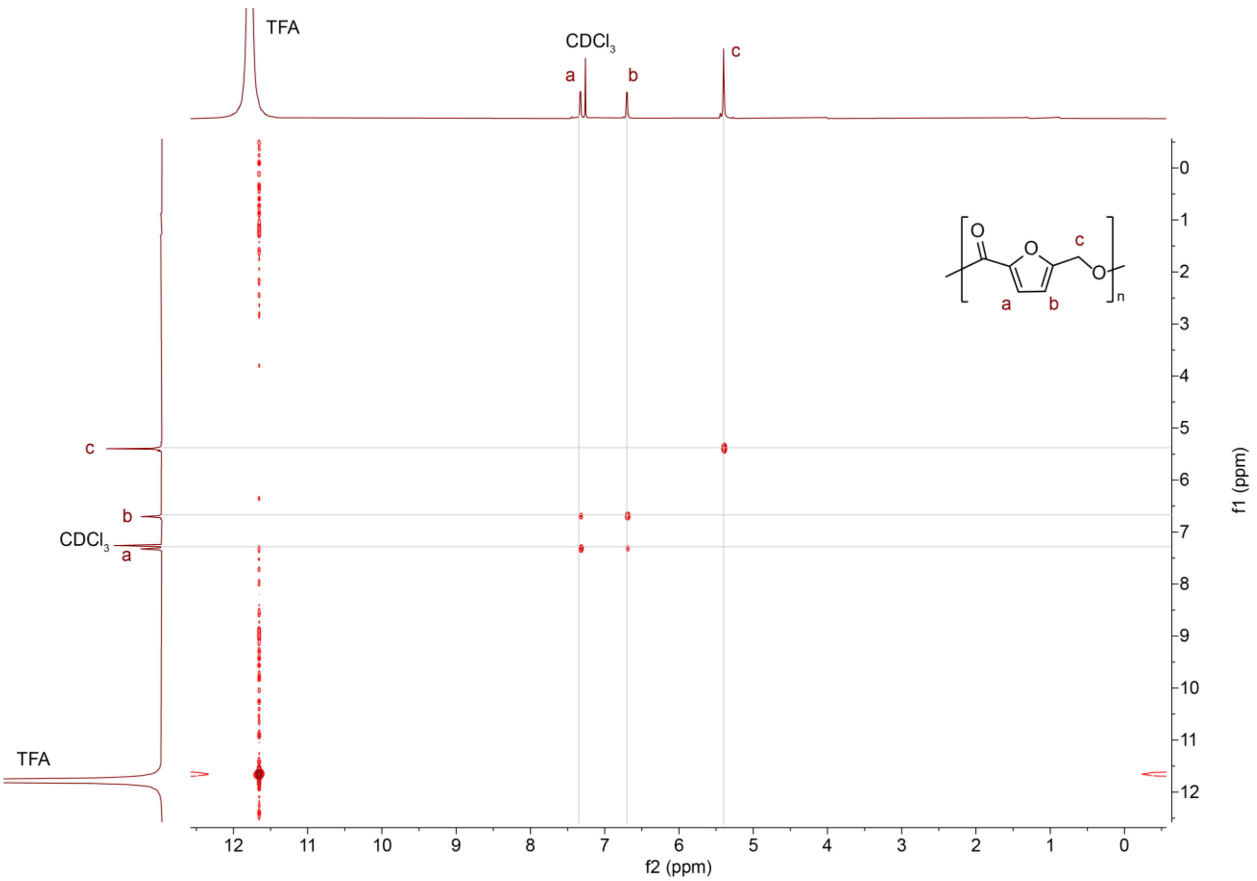

Figure S7. COSY NMR spectrum of PHMF (TFA/$CDCl_3$, 400 MHz).

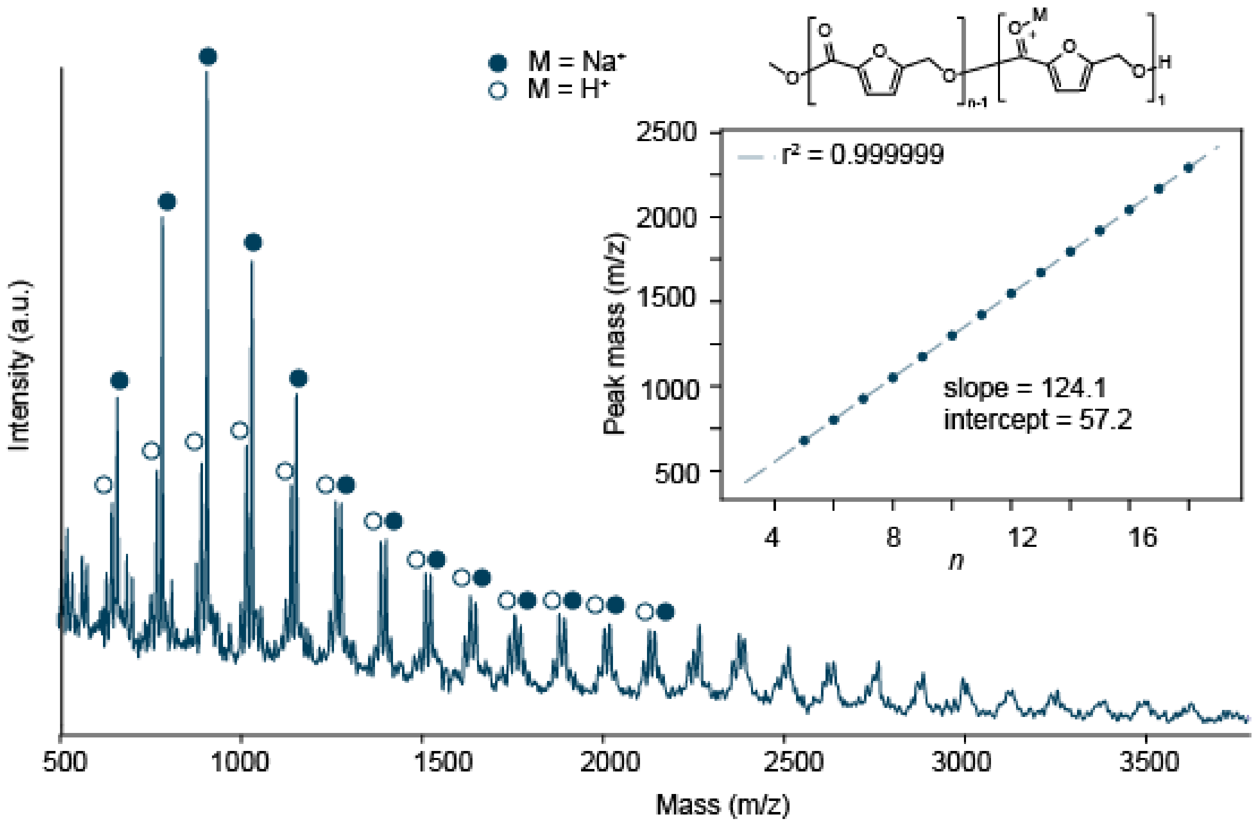

Figure S8. MALDI-TOF for low MW PHMF.

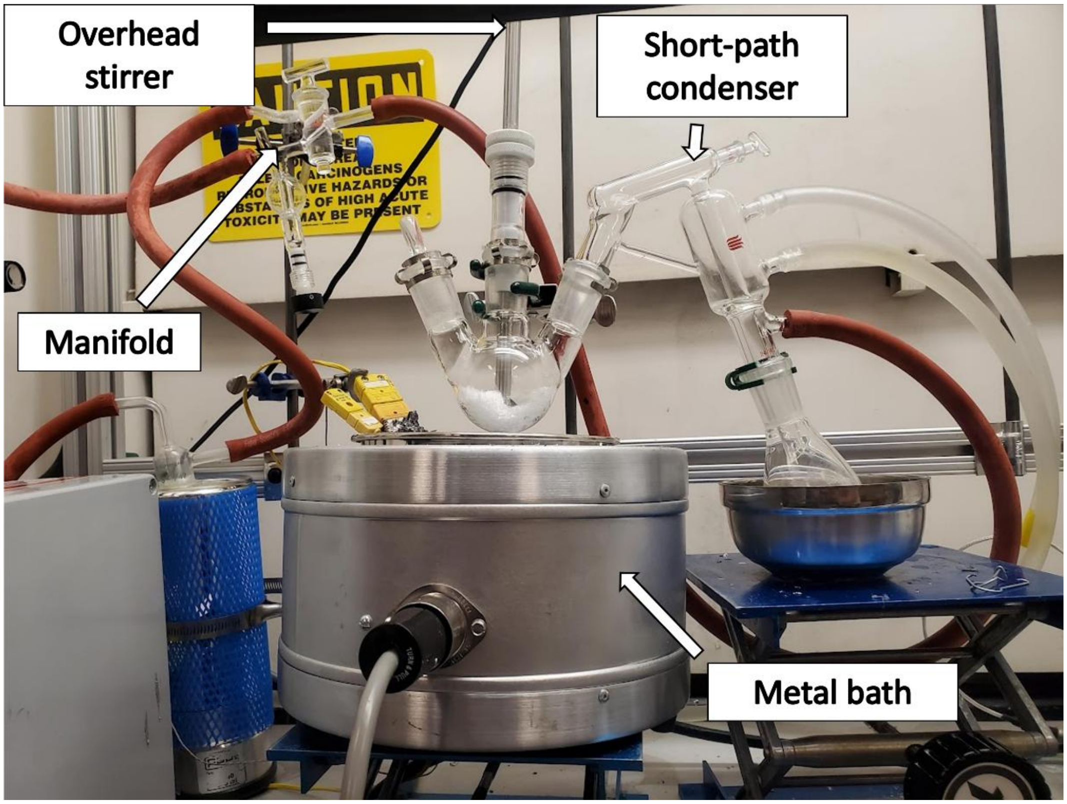

Figure S9. Polycondensation set up used in this study.

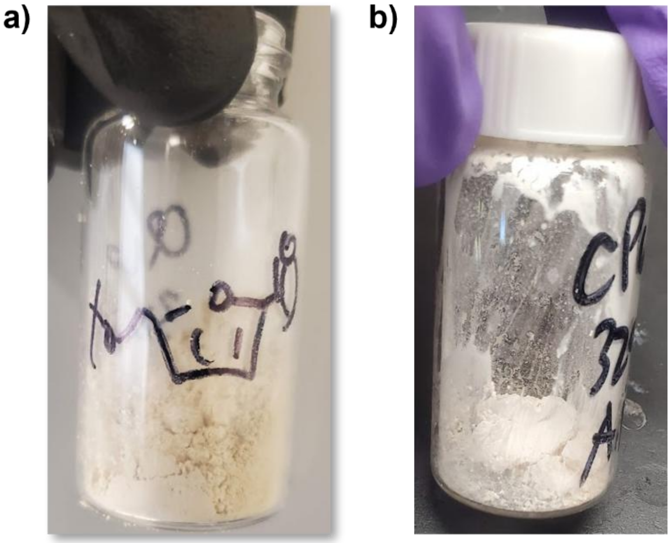

Figure S10. Color comparison of PHMF synthesized by a) melt-based polycondensation and b) solution-based polycondensation.

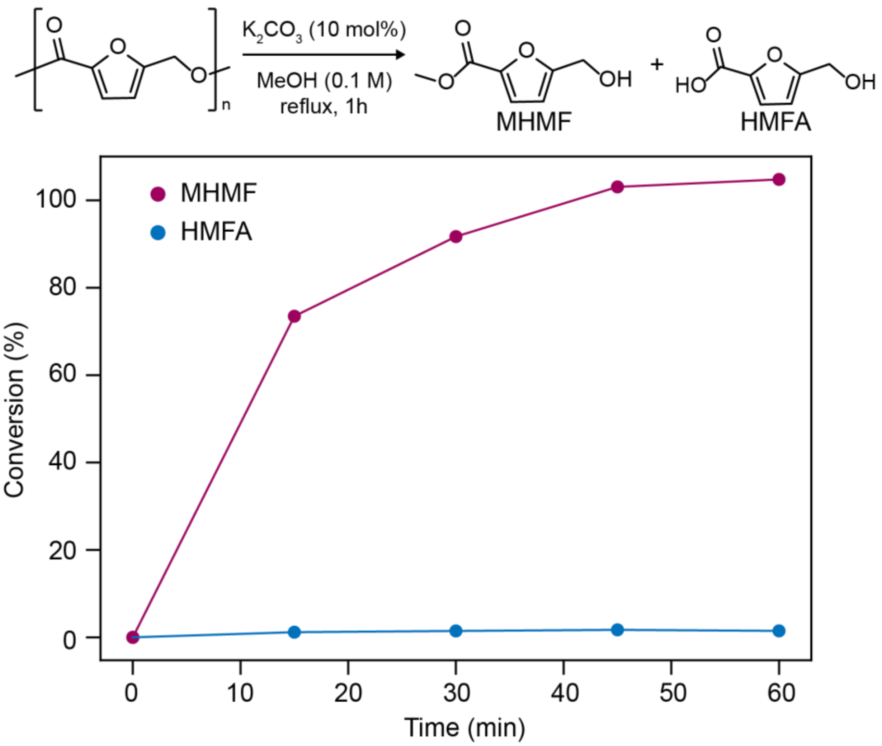

Figure S11. Depolymerization of PHMF with K_2CO_3 over time as monitored by ^1H NMR spectroscopy.

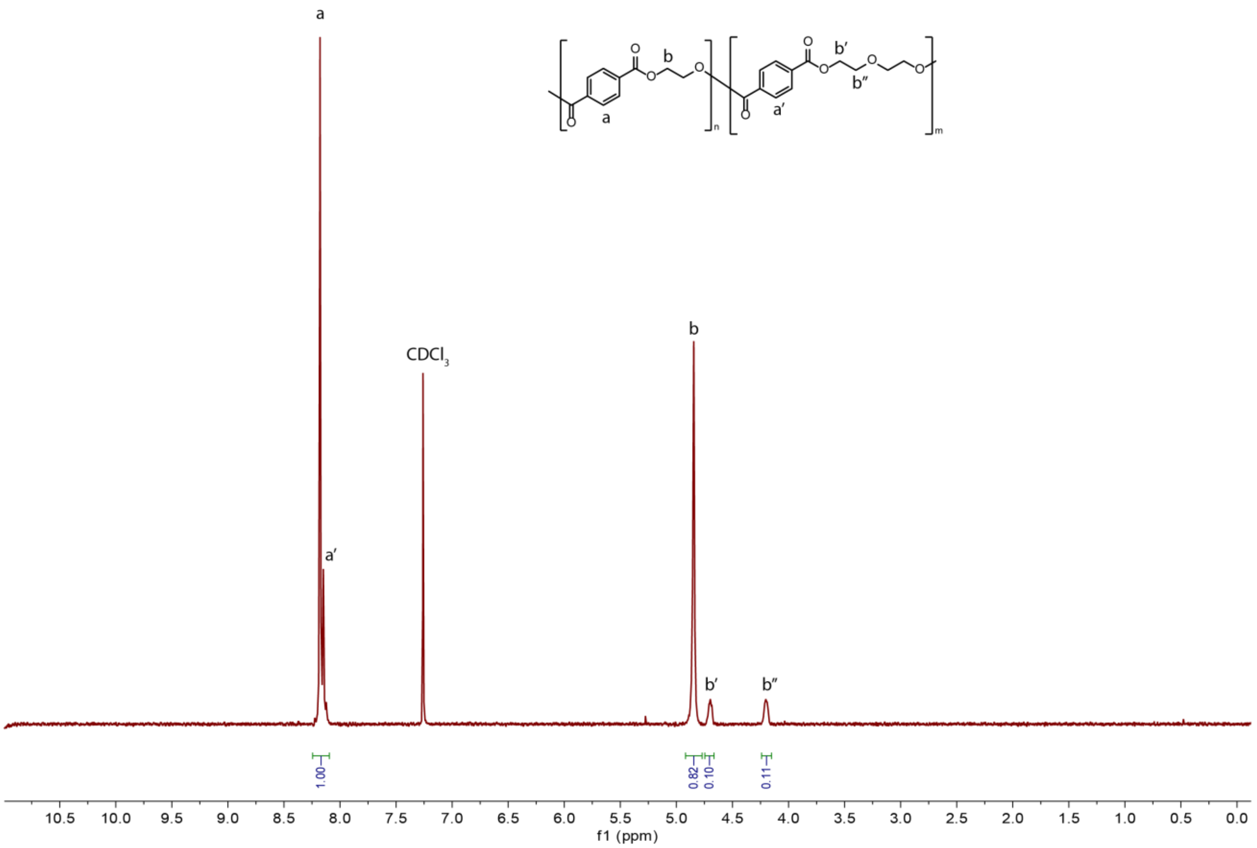

Figure S12. ^{1}H NMR spectrum of PET (TFA/$CDCl_3$, 400 MHz).

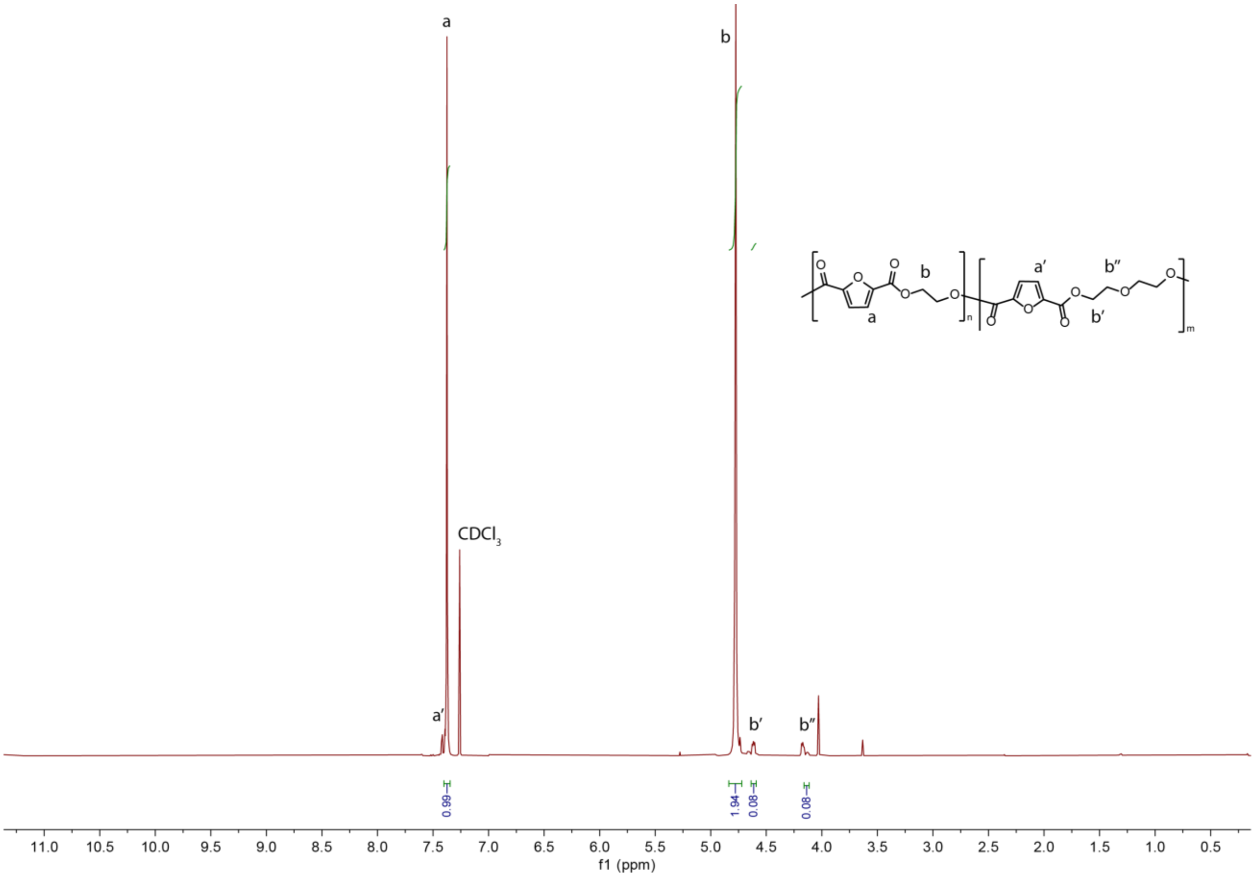

Figure S13. ^{1}H NMR spectrum of PEF (TFA/$CDCl_3$, 400 MHz).

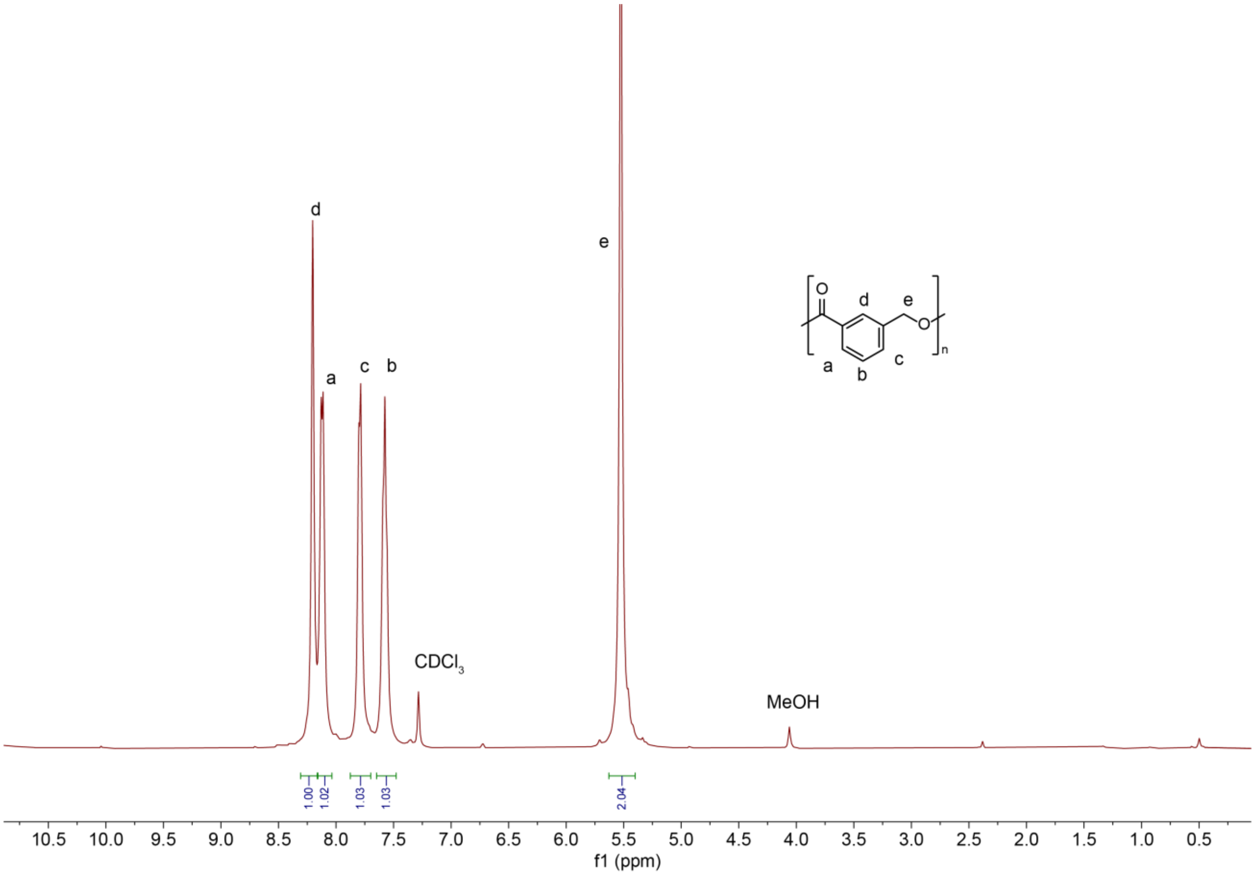

Figure S14. ^{1}H NMR spectrum of PHMB (TFA/$CDCl_3$, 400 MHz).

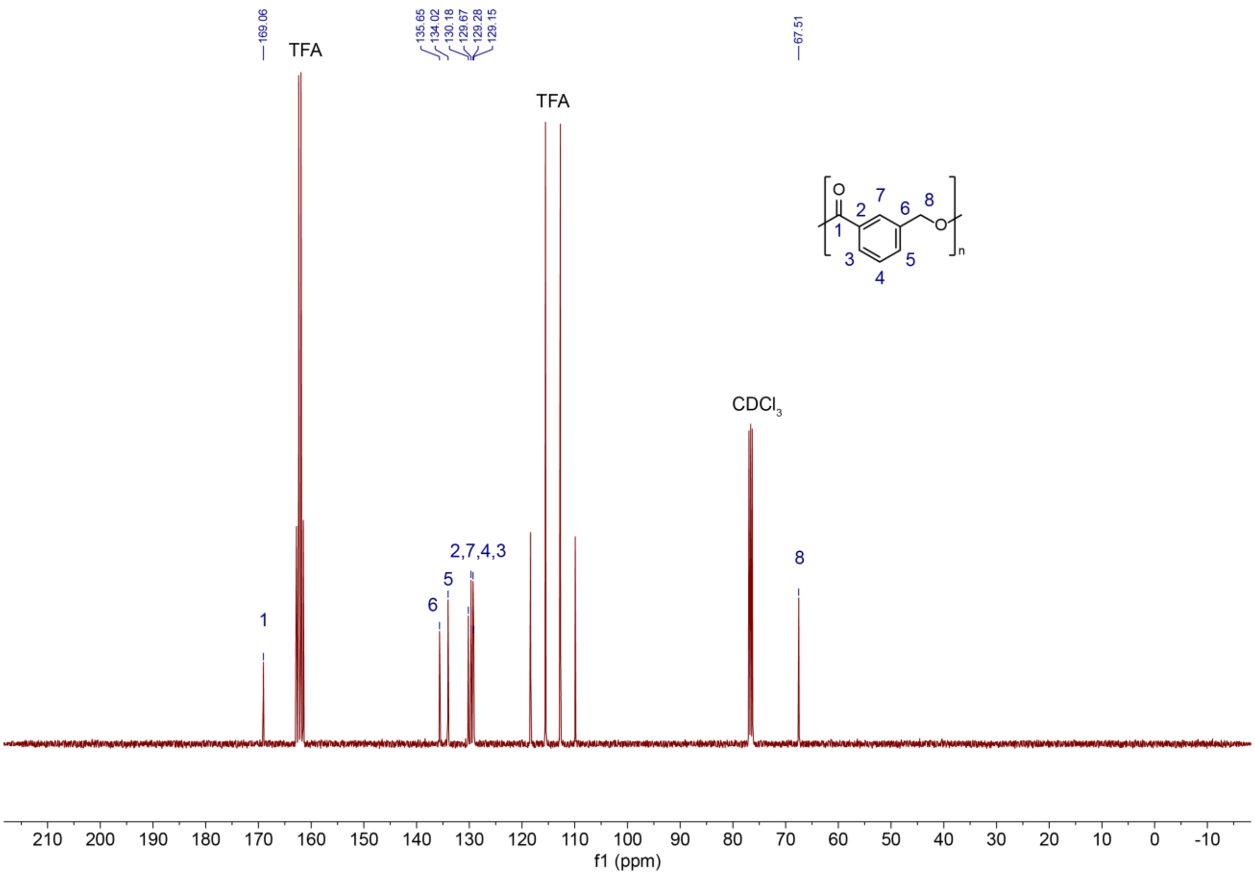

Figure S15. ^{13}C NMR spectrum of PHMB (TFA/$CDCl_3$, 101 MHz).

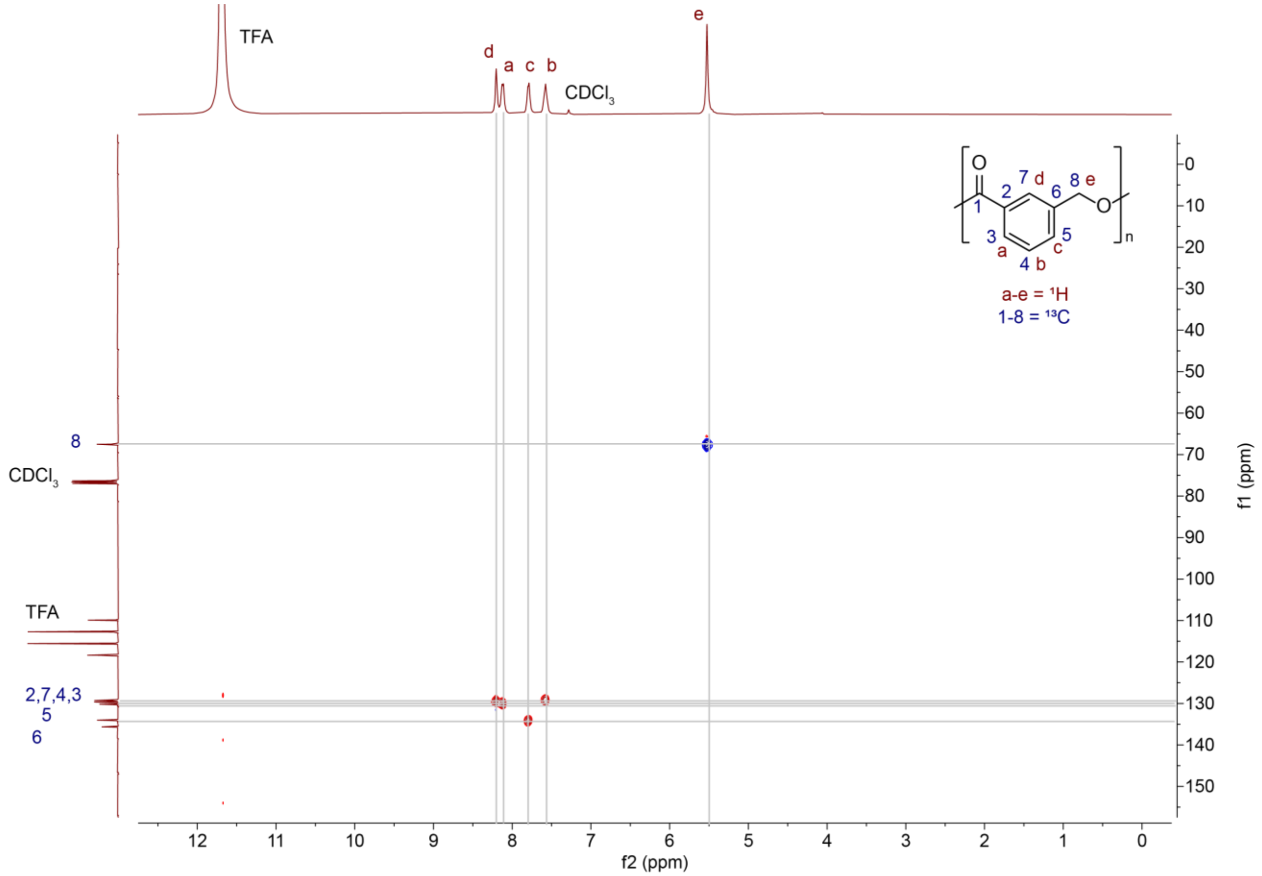

Figure S16. HSQC NMR spectrum of PHMB (TFA/$CDCl_3$, ^1H: 400 MHz, ^{13}C: 101 MHz).

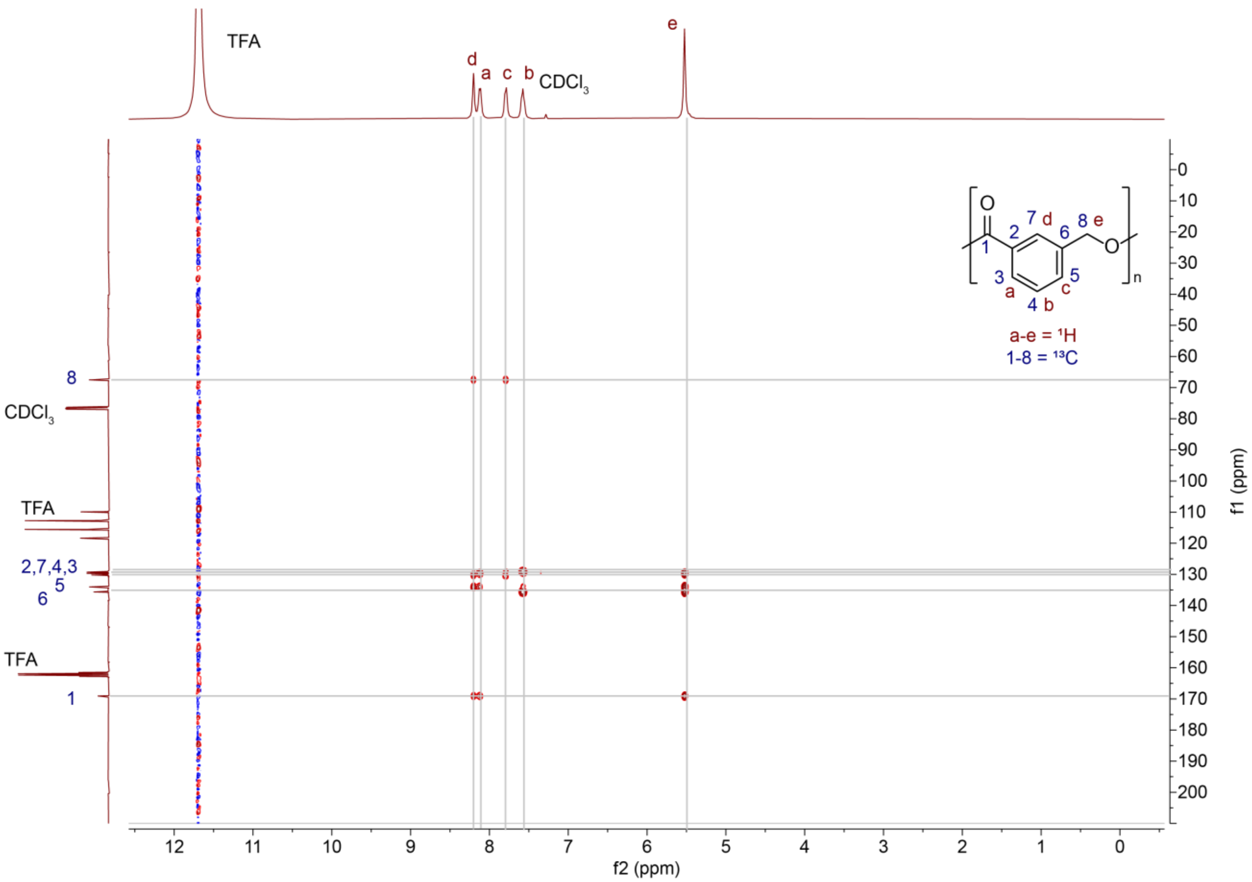

Figure S17. HMBC NMR spectrum of PHMB (TFA/$CDCl_3$, ^1H: 400 MHz, ^{13}C: 101 MHz).

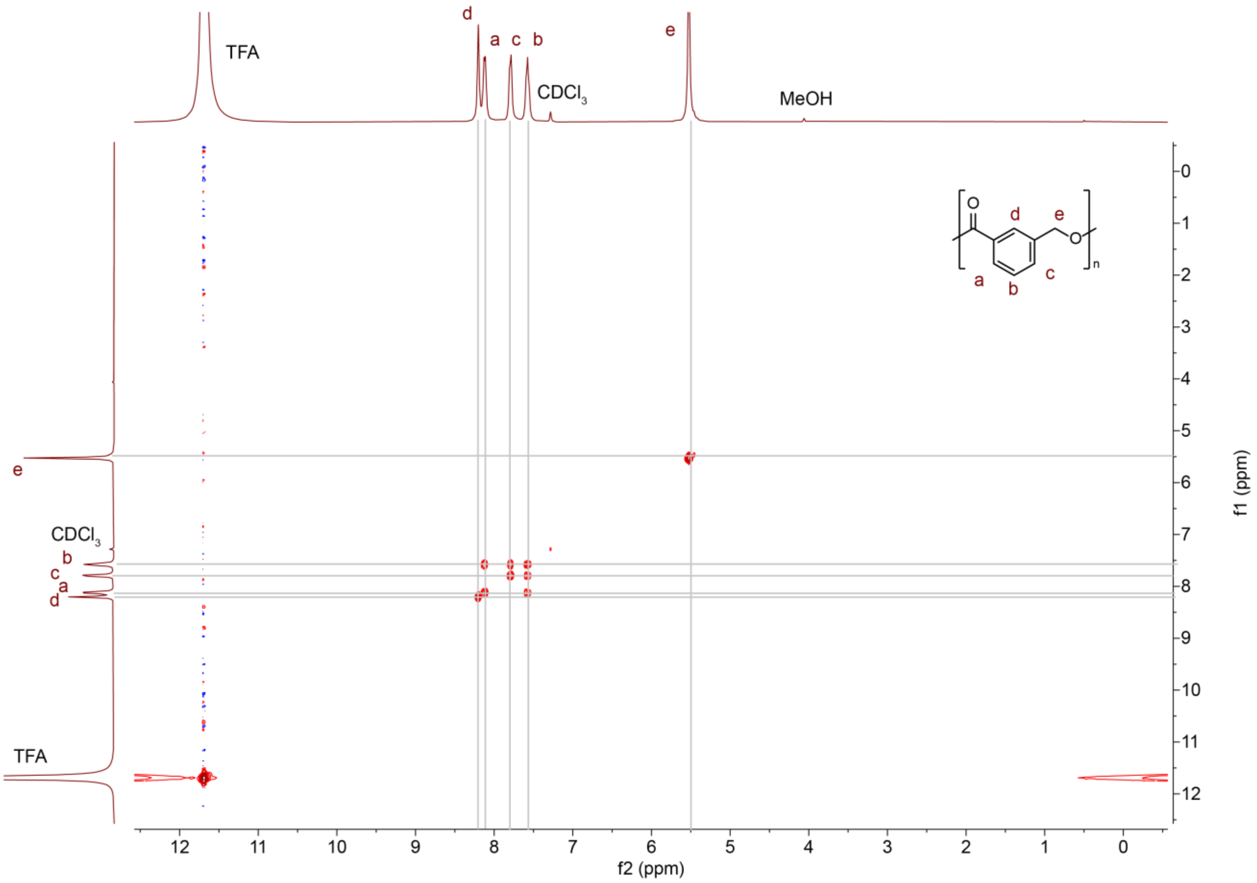

Figure S18. COSY NMR spectrum of PHMB (TFA/$CDCl_3$, 400 MHz).

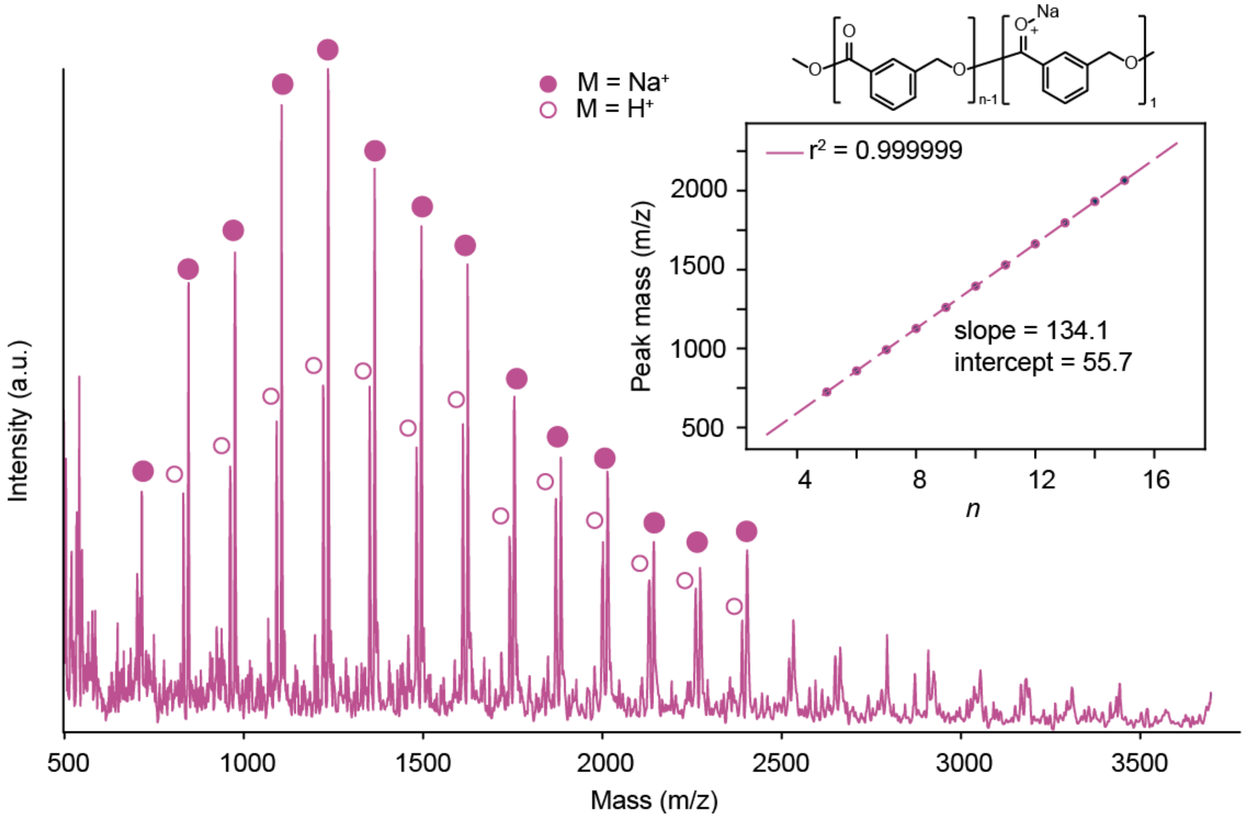

Figure S19. MALDI-TOF for low MW PHMB.

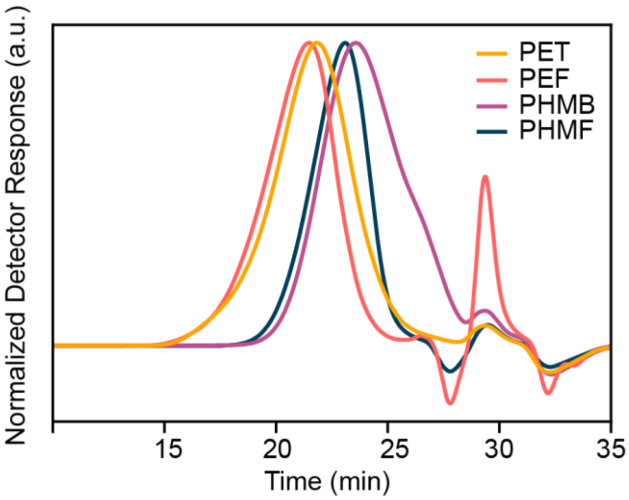

Figure S20. GPC traces in HFIP for polyesters examined in this study (t > 28 min beyond column resolution).

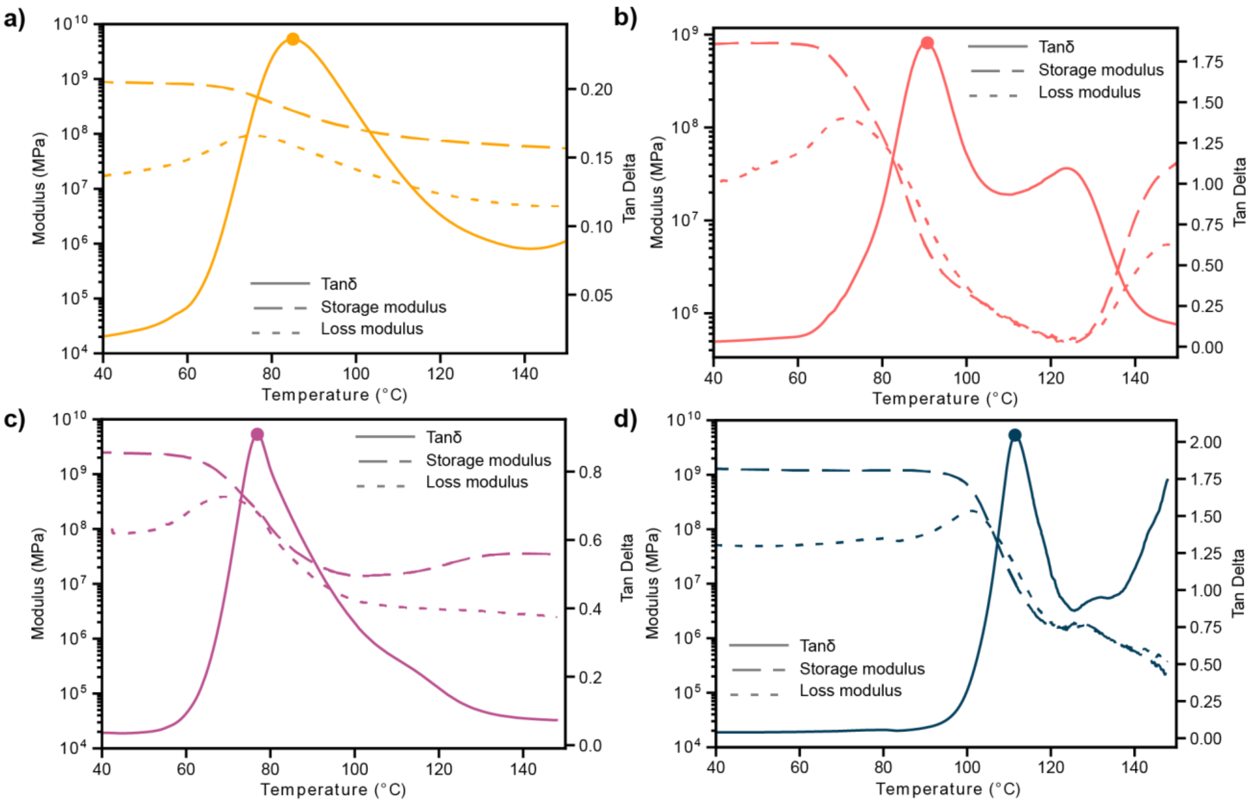

Figure S21. Dynamic mechanical analysis data for a) PET, b) PEF, c) PHMB, and d) PHMF. Solid lines represent tanδ, large, dashed lines represent storage modulus, and small dashed lines represent loss modulus.

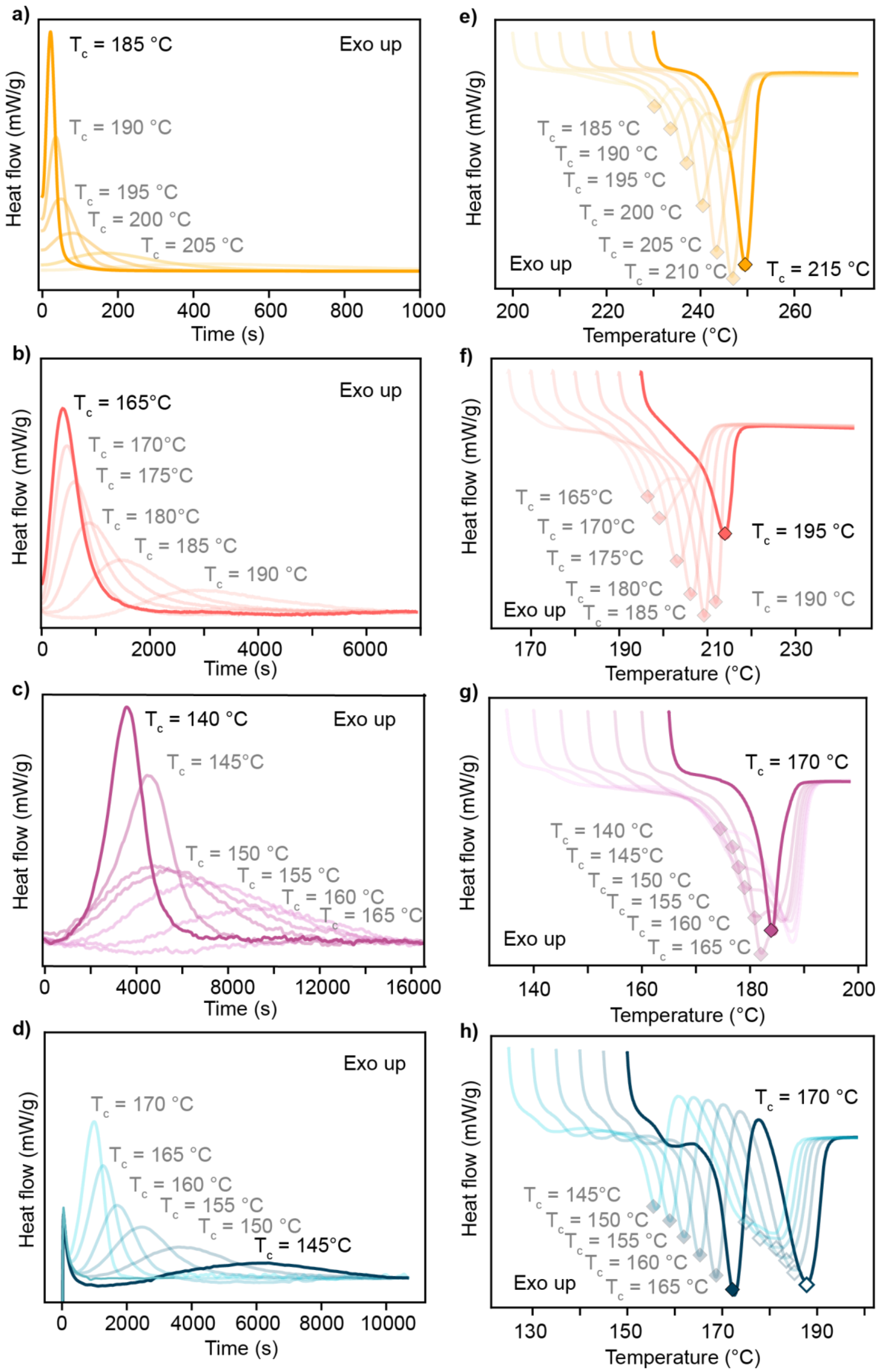
a)
T_c = 185 °C
Exo up
T_c = 190 °C
T_c = 195 °C
T_c = 200 °C
T_c = 205 °C
Heat flow (mW/g)
Time (s)
0 200 400 600 800 1000
e)
T_c = 185 °C
T_c = 190 °C
T_c = 195 °C
T_c = 200 °C
T_c = 205 °C
T_c = 210 °C
T_c = 215 °C
Exo up
Heat flow (mW/g)
Temperature (°C)
200 220 240 260
b)
T_c = 165°C
Exo up
T_c = 170°C
T_c = 175°C
T_c = 180°C
T_c = 185 °C
T_c = 190 °C
Heat flow (mW/g)
Time (s)
0 2000 4000 6000
f)
T_c = 165°C
T_c = 170°C
T_c = 175°C
T_c = 180°C
T_c = 185 °C
T_c = 195 °C
T_c = 190 °C
Exo up
Heat flow (mW/g)
Temperature (°C)
170 190 210 230
c)
T_c = 140 °C
Exo up
T_c = 145°C
T_c = 150 °C
T_c = 155 °C
T_c = 160 °C
T_c = 165 °C
Heat flow (mW/g)
Time (s)
0 4000 8000 12000 16000
g)
T_c = 170 °C
T_c = 140 °C
T_c = 145°C
T_c = 150 °C
T_c = 155 °C
T_c = 160 °C
T_c = 165 °C
Exo up
Heat flow (mW/g)
Temperature (°C)
140 160 180 200
d)
Exo up
T_c = 170 °C
T_c = 165 °C
T_c = 160 °C
T_c = 155 °C
T_c = 150 °C
T_c = 145°C
Heat flow (mW/g)
Time (s)
0 2000 4000 6000 8000 10000
h)
T_c = 170 °C
T_c = 145°C
T_c = 150 °C
T_c = 155 °C
T_c = 160 °C
T_c = 165 °C
Exo up
Heat flow (mW/g)
Temperature (°C)
130 150 170 190

Figure S22. Results from isothermal crystallization experiments of polyesters measured by DSC. Rate of crystallization as measured by heat flow over time at T_c for a) PET, b) PEF, c) PHMB, and d) PHMF. Changes in melting point as measured by heating to T_m + 30 °C for e) PET, f) PEF, g) PHMB, and d) PHMF. Results for highest T_c are bolded.

3. Supporting tables

Table S1. Optimization of small-scale polycondensation of MHMF.

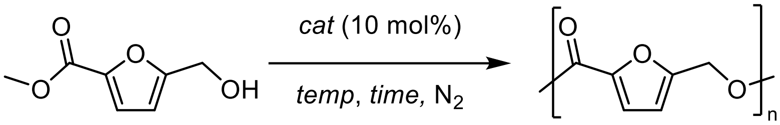

Entry	Temperature (°C)	Time (h)	Catalyst	Conversion (%)[a]	Crude Yield (%)	$M_{n,NMR}$ (kDa)
1	180	16	-	35%	-	0.5
2	180	4	$Ca(Ac)_2$	33%	17%	5.4
3	180	4	Sb_2O_3	38%	16%	7.0
4	180	4	$Zn(Ac)_2$	41%	31%	0.3
5	180	4	$Ti(i\text{-}PrO)_4$	73%	59%	0.6
6	180	4	$(Oct)_2SnO$	92%	59%	18.1
7	160	18	$(Oct)_2SnO$	91%	73%	15.3
8	140	3	$(Oct)_2SnO$	89%	58%	18.1
9	140	9	$(Oct)_2SnO$	95%	76%	18.6

[a] Determined by crude conversion by NMR spectroscopy. [b] Determined by crude mass after precipitation. [c] Determined by end-group analysis by NMR spectroscopy. Conditions: MHMF (50 mg, 321 µmol), *catalyst* (32 µmol, 5 mol%), *temp*, *time*

Table S2. Polymerization of PHMF at increased scale.

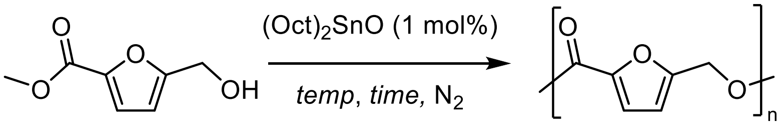

Entry	Reaction conditions (°C)	Overhead stirring	Yield (%)	$M_{n,NMR}$ (kDa)
1	1) 140 °C, 18 h, N_2	None	19%	14.4
2	1) 140 °C, 2 h, N_2<br>2) 160 °C, 2 h, N_2<br>3) 180 °C, 2 h, N_2<br>4) 180 °C, 18 h, vacuum	50 rpm	52%	9.8
3	1) 140 °C, 16 h, N_2<br>2) 160 °C, 2 h, N_2<br>3) 180 °C, 2 h, N_2<br>4) 180 °C, 18 h, vacuum	50 rpm	73%	19.2

[a] Determined by crude conversion by NMR spectroscopy. [b] Determined by crude mass after precipitation. [c] Determined by end-group analysis by NMR spectroscopy. Conditions: MHMF (50 mg, 321 µmol), *catalyst* (32 µmol, 5 mol%), *temp*, *time*

Table S3. Preliminary oligomerization of MHMF

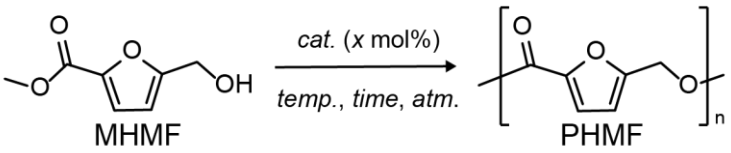

Entry	Temp. (°C)	Time (h)	Cat.	Conv. (%)[a]	Yield (%)[b]	$M_{n,NMR}$ (kDa)[c]
1	180	4	Ti(i-PrO)$_4$	73%	–	–
2	180	4	Zn(Ac)$_2$	41%	–	–
3	180	4	Sb_2O_3	38%	–	–
4	180	4	Ca(Ac)$_2$	33%	–	–
5	180	4	(Oct)$_2$SnO	92%	–	–
6	160	18	(Oct)$_2$SnO	91%	–	–
7	160	18	Ti(i-PrO)$_4$	65%	–	–
8	140	3	(Oct)$_2$SnO	89%	53%	1.1
9	140	6	(Oct)$_2$SnO	93%	62%	1.7
10	140	9	(Oct)$_2$SnO	95%	68%	2.3

Conditions: MHMF (10 mg), catalyst (1 equiv), temp, time, flowing N_2. [a] Conversion determined by ^{1}H NMR spectroscopy in $CDCl_3$/TFA. [b] Isolated yield determined by precipitation in methanol. [c] $M_{n,NMR}$ determined by end group analysis of isolated PHMF via ^{1}H NMR spectroscopy in $CDCl_3$/TFA

Table S4. Condition screen for methanolysis of PHMF.

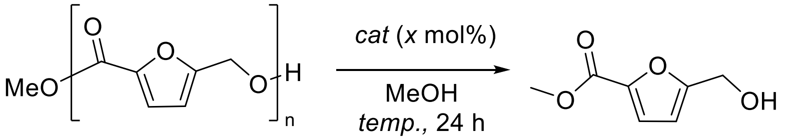

Entry	Temperature (°C)	Catalyst	x (mol %)	Conc. (M)	Conversion (%)
1	80	-	-	0.08	2%
2	80	$FeCl_3$	0.5	0.08	3%
3	80	DMAc	1.5	0.12	1%
4	80	Et_3N	1	0.08	2%
5	80	H_2SO_4	5	0.03	11%
6	80	$ZnCl_2$	3.5	0.03	2%
7	80	Zn(Ac)$_2$	1.5	0.04	61%
8	80	K_2CO_3	2.5	0.06	61% (39%)[b]
9	60	-	-	0.03	0%
10	60	Zn(Ac)$_2$	1	0.02	31%
11	80	Zn(Ac)$_2$	0.5	0.06	58%
12	80	Zn(Ac)$_2$	0.1	0.10	45%
13	80	K_2CO_3	0.25	0.08	66% (33%)[b]

Conditions: PHMF (10 mg), catalyst, MeOH, temp., 24 h. [a] Conversion determined by ^{1}H NMR spectroscopy with a toluene standard (1 μL). [b] Conversion to HMFA in parentheses.

Table S5. Summary of the experimental polymer properties examined in this work.

Polymer	PHMF	PHMB	PEF	PET
$M_{n,GPC}$ (kDa)	13.7	7.0	15.7	21.7
$M_{w,GPC}$ (kDa)	31.5	18.9	53.4	52.1
Đ	2.3	2.7	3.4	2.4
DP	110	52	86	113
$T_{g,DSC}$ (°C)	83	67	80	74
$T_{g,DMA}$ (°C)	112	77	90	85
T_m (°C)	188	190	215	249
$T_{d,5\%}$ (°C)	312	379	358	395
T_m° (°C)	236	190	238	283
$t_{1/2}$	912	4469	147	284
$T_{1\rho}$ (ms)	35.9	54.7	17.8	8.7
T_{CH} (ms)	1.16	1.13	1.39	1.28

Table S6. Molecular dynamics equilibration procedure.

Step	Ensemble	Time (ns)	Temperature (K)	Pressure (bar)
1	NVT	1	650	-
2	NVT	1	300	-
3	NPT	5	300	1000
4	NVT	1	650	-
5	NVT	1	300	-
6	NPT	5	300	5000
7	NVT	1	650	-
8	NVT	1	300	-
9	NPT	5	300	10,000
10	NVT	1	650	-
11	NVT	1	300	-
12	NPT	5	300	20,000
13	NVT	1	650	-
14	NVT	1	300	-
15	NPT	5	300	30,000
16	NVT	1	650	-
17	NVT	1	300	-
18	NPT	5	300	40,000
19	NVT	1	650	-
20	NVT	1	300	-
21	NPT	5	300	50,000
22	NVT	1	650	-
23	NVT	1	300	-
24	NPT	5	300	60,000

25	NVT	0.1	650	-
26	NVT	0.1	300	-
27	NPT	0.1	300	50,000
28	NVT	0.1	650	-
29	NVT	0.1	300	-
30	NPT	0.1	300	25,000
31	NVT	0.1	650	-
32	NVT	0.1	300	-
33	NPT	0.1	300	10,000
34	NVT	0.1	650	-
35	NVT	0.1	300	-
36	NPT	10	300	1

Table S7. Computed properties from molecular dynamics simulations compared to reported experimental values (references in paratheses).

Polymer	PHMF	PHMB	PEF	PET
DP	100	100	100	100
d_{sim} (g/cm^3)	1.24	1.36	1.38	1.29
d_{exp} (g/cm^3)	–	–	1.43(13)	1.33(13)
$C_{\infty,\mathrm{sim}}$	7.2	6.8	4.8	5.2
$C_{\infty,\mathrm{exp}}$	–	–	–	4.1-5.8(82–84)

4. Supporting equations

4.1. Avrami analysis

The Avrami equation (Eq. S1) models the crystallization of a solid from the melt with a nucleation and growth mechanism:

$$X(t) = 1 - e^{-(kt)^n} \qquad \text{(Eq. S1)}$$

Where *X(t)* is the extent of crystallization, k is the Avrami rate constant, and n is the Avrami exponent. The Avrami exponent serves as an indicator into the nature of the nucleation and growth type process and typically ranges between 1 and 4. At greater values of n, the crystallization is expected to occur with higher degrees of dimensionality.

4.2. Variable contact time-cross polarization-magic angle spinning (VCT-CP-MAS) NMR spectroscopy analysis

VCT-CP-MAS NMR spectroscopy was performed on amorphous polymer samples. Each sample was subjected to VCT-CP-MAS with 32 different contact pulse steps between 0.01 and 25 ms. After the measurement was complete, the intensity of the carbonyl peak was fit to using the following expression to extract quantitative information about the relaxation and mobility of the carbonyl),

$$I = A \frac{\left(\exp\left(-\frac{t}{T_{1\rho}}\right) - \exp\left(-\frac{t}{T_{1CH}}\right)\right)}{1 - \frac{T_{1\rho}}{T_{1CH}}} \qquad \text{(Eq. S2)}$$

where t is the contact pulse time, I is the observed intensity, T_{1CH} is the time constant of the magnetization buildup, and $T_{1\rho}$ is the time constant for magnetization decay. The parameters extracted from each polyester are as follows:

Entry	Polymer	$T_{1\rho}$ (ms)	T_{1CH} (ms)
1	PET	8.7	1.28
2	PEF	17.8	1.39
3	PHMB	54.7	1.13
4	PHMF	35.9	1.16

4.3. Characteristic ratio

The characteristic ratio was calculated via the following relationship:

$$C_\infty = \frac{\langle R^2 \rangle}{nl^2} \qquad \text{(Eq. S3)}$$

Where C_∞ is the characteristic ratio, $\langle R^2 \rangle$ is the mean-squared extended chain length, n is the number of repeat units, and l is the segment length.

5. Supporting References

(12) Knoop, R. J. I.; Vogelzang, W.; Haveren, J. V.; Es, D. S. V. High Molecular Weight Poly(Ethylene-2,5-Furanoate); Critical Aspects in Synthesis and Mechanical Property Determination. *J. Polym. Sci. Part Polym. Chem.* **2013**, *51* (19), 4191–4199. https://doi.org/10.1002/POLA.26833.

(43) Palermo, E. F.; Chiu, J. Critical Review of Methods for the Determination of Purity by Differential Scanning Calorimetry*. *Thermochim. Acta* **1976**, *14* (1), 1–12. https://doi.org/10.1016/0040-6031(76)80053-6

(44) Gubbels, E.; Heitz, T.; Yamamoto, M.; Chilekar, V.; Zarbakhsh, S.; Gepraegs, M.; Köpnick, H.; Schmidt, M.; Brügging, W.; Rüter, J.; Kaminsky, W. Polyesters. In *Ullmann's Encyclopedia of Industrial Chemistry*; John Wiley & Sons, Ltd, 2018; pp 1–30. https://doi.org/10.1002/14356007.a21_227.pub2

(75) Abbott, L. J.; Hart, K. E.; Colina, C. M. Polymatic: A Generalized Simulated Polymerization Algorithm for Amorphous Polymers. *Theor. Chem. Acc.* **2013**, *132* (3), 1–19. https://doi.org/10.1007/s00214-013-1334-z.

(76) Rorrer, N. A.; Notonier, S. F.; Knott, B. C.; Black, B. A.; Singh, A.; Nicholson, S. R.; Kinchin, C. P.; Schmidt, G. P.; Carpenter, A. C.; Ramirez, K. J.; Johnson, C. W.; Salvachúa, D.; Crowley, M. F.; Beckham, G. T. Production of β-Ketoadipic Acid from Glucose in Pseudomonas Putida KT2440 for Use in Performance-Advantaged Nylons. *Cell Rep. Phys. Sci.* **2022**, *3* (4), 100840. https://doi.org/10.1016/J.XCRP.2022.100840.

(81) Serum, E. M.; Sutton, C. A.; Renner, A. C.; Dawn, D.; Sibi, M. P. New AB Type Monomers from Lignocellulosic Biomass. *Pure Appl. Chem.* **2019**, *91* (3), 389–396. https://doi.org/10.1515/pac-2018-0913.

(82) Tonelli, A. E. Conformational Characteristics of Poly(Ethylene Phthalate)s. *J. Polym. Sci. Part B Polym. Phys.* **2002**, *40* (12), 1254–1260. https://doi.org/10.1002/polb.10189.

(83) Youk, J. H.; Jo, W. H.; Yoo, D. I. Molecular Dimensions of Poly(Ethylene Isophthalate) and Poly(Ethylene Naphthalene-2,6-Dicarboxylate). *Polym. Bull.* **1997**, *39* (2), 257–263. https://doi.org/10.1007/s002890050146.

(84) Cusano, I.; Campagnolo, L.; Aurilia, M.; Costanzo, S.; Grizzuti, N. Rheology of Recycled PET. *Materials* **2023**, *16* (9), 3358. https://doi.org/10.3390/ma16093358.

(85) Zekriardehani, S.; Jabarin, S. A.; Gidley, D. R.; Coleman, M. R. Effect of Chain Dynamics, Crystallinity, and Free Volume on the Barrier Properties of Poly(Ethylene Terephthalate) Biaxially Oriented Films. *Macromolecules* **2017**, *50* (7), 2845–2855. https://doi.org/10.1021/acs.macromol.7b00198.

(86) McGonigle, E.-A.; Liggat, J. J.; Pethrick, R. A.; Jenkins, S. D.; Daly, J. H.; Hayward, D. Permeability of N2, Ar, He, O2 and CO2 through Biaxially Oriented Polyester Films — Dependence on Free Volume. *Polymer* **2001**, *42* (6), 2413–2426. https://doi.org/10.1016/S0032-3861(00)00615-7.
